# Phosphorylation of nuclear Tau is modulated by distinct cellular pathways

**DOI:** 10.1038/s41598-018-36374-4

**Published:** 2018-12-07

**Authors:** Giorgio Ulrich, Agnese Salvadè, Paul Boersema, Tito Calì, Chiara Foglieni, Martina Sola, Paola Picotti, Stéphanie Papin, Paolo Paganetti

**Affiliations:** 1 Laboratory for Biomedical Neurosciences, Neurocenter of Southern Switzerland, Ente Cantonale Ospedaliero, Torricella-Taverne, Switzerland; 2 Institute of Molecular Systems Biology, Department of Biology, ETHZ, Zurich, Switzerland; 3 Department of Biomedical Sciences and Padova Neuroscience Center, University of Padova, Padova, Italy

## Abstract

Post-translational protein modification controls the function of Tau as a scaffold protein linking a variety of molecular partners. This is most studied in the context of microtubules, where Tau regulates their stability as well as the distribution of cellular components to defined compartments. However, Tau is also located in the cell nucleus; and is found to protect DNA. Quantitative assessment of Tau modification in the nucleus when compared to the cytosol may elucidate how subcellular distribution and function of Tau is regulated. We undertook an unbiased approach by combing bimolecular fluorescent complementation and mass spectrometry in order to show that Tau phosphorylation at specific residues is increased in the nucleus of proliferating pluripotent neuronal C17.2 and neuroblastoma SY5Y cells. These findings were validated with the use of nuclear targeted Tau and subcellular fractionation, in particular for the phosphorylation at T_181_, T_212_ and S_404_. We also report that the DNA damaging drug Etoposide increases the translocation of Tau to the nucleus whilst reducing its phosphorylation. We propose that overt phosphorylation of Tau, a hallmark of neurodegenerative disorders defined as tauopathies, may negatively regulate the function of nuclear Tau in protecting against DNA damage.

## Introduction

Proteinopathies represent a large spectrum of human disorders caused by proteins with a cytotoxic gain of function or the failure to perform a normal activity, both due to abnormal conformation and modification^1^. In their pathogenic forms, these proteins hold the predisposition to self-assemble into toxic soluble oligomers or insoluble aggregates. Given that cellular protein clearance is less efficient for multimeric or aggregated protein assemblies, a gradual accumulation and the formation of large deposits such as those typical for progressive neurodegenerative disorders may occur^2^. This is further accelerated by aging, which correlates with proteostasis defects, mostly due to a decline in protein clearance^3^. Other liabilities are increased protein production, abnormal post-translational modification, or changes in the amino acid sequence of the protein in genetic variants causing hereditary disease forms^4^. The aging brain may be concerned by the co-existence of distinct proteinopathies such as those involving Tau in neurofibrillar tangles and β-amyloid plaques in Alzheimer’s disease, or α-synuclein in Lewi bodies of Parkinson’s disease^5,6^. Then again, distinct proteinopathies may cause clinically similar disorders as it is the case for the deposition of aberrant forms of Tau, FUS or TDP-43 in the ALS/FTD disease spectrum^7^. Tauopathies, both in sporadic and familial forms of frontotemporal dementia with parkinsonism-17 caused by mutations in the Tau gene (*MAPT*), are characterized by Tau assembled in highly ordered paired helical filaments within neuronal cells^8,9^.

Tau was originally isolated as a microtubule-associated protein^10^. Its primary structure covers domains with distinct characteristics typical of a scaffold protein: an amino-terminal projection sequence followed by a proline-rich sequence domain, a microtubule-binding region and a carboxy-terminal tail. In its unbound state, Tau is described as highly soluble, heath-stable, unfolded protein. Tau binding to microtubules leads to a conformational switch with the negatively charged projection domain dissociating from the positively charged microtubule-binding domain. Tau links microtubules to other binding partner such as motor complexes or cellular membranes^11^, a process that is regulated by post-translational modifications^9,12^. Tau is post-translationally modified by proteolysis, acetylation, methylation, glycosylation and phosphorylation. This latter is the prevalent modification with more than eighty potential sites at serines, threonines and tyrosines. About twenty sites contribute to the normal function of Tau, but increased phosphorylation at these or at additional sites occurs during early development and is present in pathological lesions^13^. Phosphorylation within or outside the microtubule domain affects Tau association to microtubules. This may disturb microtubule-mediated axonal transport or increase free Tau, the probability of fibril formation^14^ or its secretion as a critical event for cell-to-cell spreading of Tau lesions in the brain^15,16^. Microtubule-dissociated Tau may also locate to other cellular compartments^11,17,18^. Toxic insults induce abnormal Tau distribution, as it is the case for β-amyloid-mediated re-location of Tau from the axonal to the somatodendritic compartment of neurons^19^. Distinct cellular locations of Tau may be associated to different post-translational modifications^9,12,20^ and different post-translational modifications of Tau may be associated to different functions, e.g. at the neuronal synapse^21,22^. Intriguingly, Tau is also found in the nucleus *in vitro*^23–27^ and *in vivo*^28^. Tau interacts with RNA and DNA^29–32^ and appears to protect DNA from denaturation and radicals^33–36^. Binding of DNA by Tau is linked to its dephosphorylated form^37^.

In our study, we revisited the hypothesis that nuclear Tau is characterized by a distinct post-translational modification. Using a set of parallel approaches to characterize Tau modification in the nucleus, we found increased phosphorylation at distinct sites for nuclear localized Tau and identified pharmacological modulators that differentially affect the subcellular location and modification of Tau.

## Results

### Cellular distribution of Tau by bimolecular fluorescent complementation

**Figure 1:**
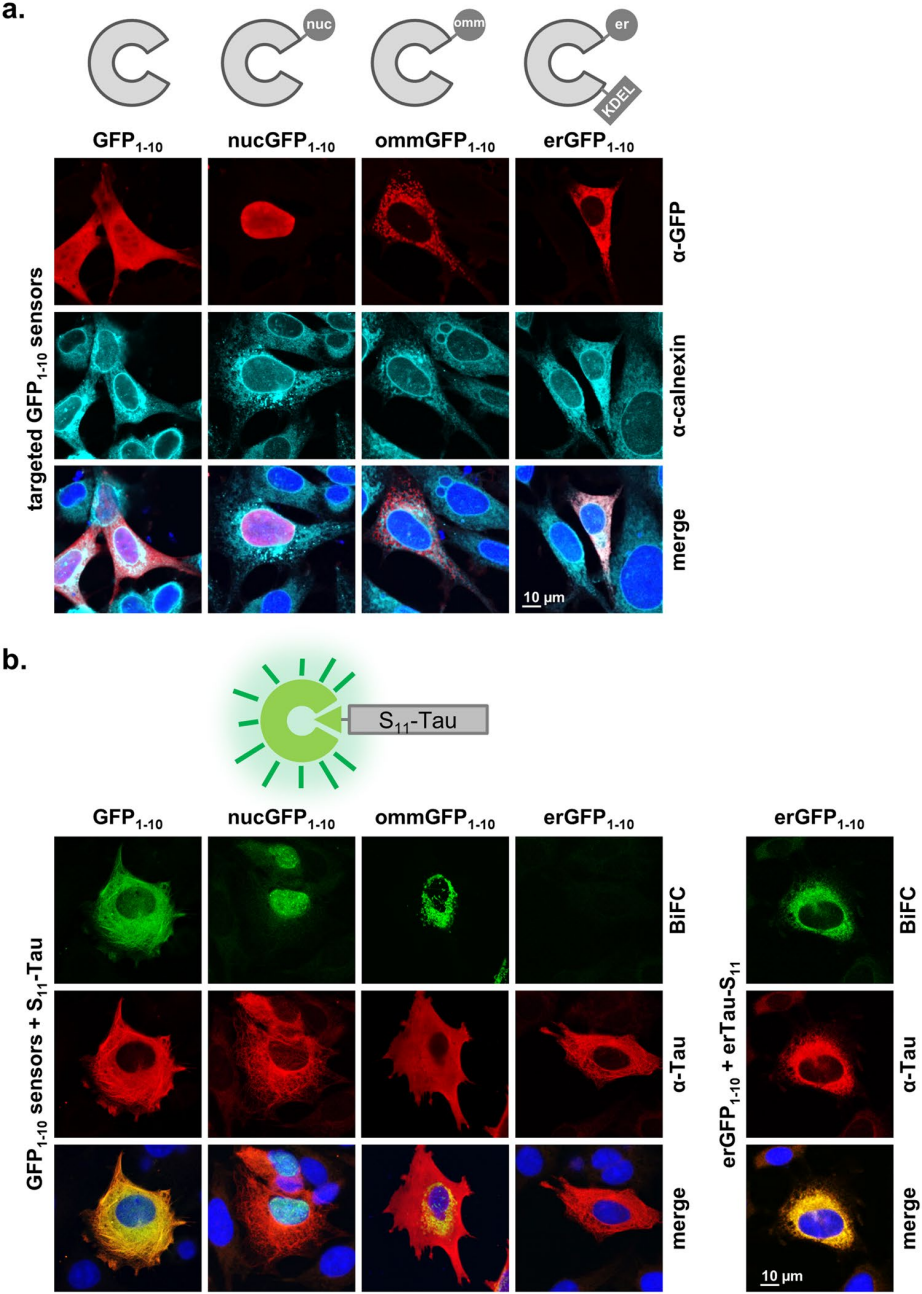
Targeted GFP_1–10_ sensors reveal subcellular pools of Tau. (**a**) The subcellular distribution of the indicated GFP_1–10_ sensors in transiently transfected mouse C17.2 cells is shown by confocal microscopy upon immune staining of PFA-fixed cells with an anti-GFP antibody (upper row, in red). The cells are counter-stained with the ER-marker calnexin (middle row, in cyan) and the nuclear stain DAPI (shown in the merged images, bottom row, in blue). (**b**) The GFP_1–10_ sensors were then co-transfected with S_11_-Tau, which reveals by biFC biofluorescence (upper row, in green) microtubule-associated Tau with GFP_1–10_, nuclear Tau with nucGFP_1–10_, and mitochondria-associated Tau with ommGFP_1–10_. No biFC is obtained with the erGFP_1–10_ sensor, unless Tau is targeted to the ER lumen (erTau-S_11_, column on the far right). The cells are counter-stained for total human Tau with the Tau13 antibody (middle row, in red) and DAPI (merged images, bottom row, in blue).

Bimolecular fluorescence complementation (biFC) represents a relatively simple technology to reveal subcellular protein location^38^. We adapted biFC to investigate the presence of Tau in specific sublocations within cultured cells. Here, we generated GFP_1–10_ sensors targeted to the nucleus, to the lumen of the endoplasmic reticulum (ER) or to the mitochondrial outer membrane (OMM). For the nuclear targeting sequence fused at the amino-terminus of GFP_1–10_ (nucGFP_1–10_), we took advantage of a well-described artificial targeting peptide carrying three times a nuclear targeting sequence derived from the SV40 large T antigen^39^. For the erGFP_1–10_ sensor, we fused the signal peptide of human calreticulin at the amino-terminus of the sensor and added the ER retention signal KDEL at its carboxy-terminus (Fig. 1a). The ommGFP_1–10_ sensor was generated as described^18,40^. The correct cellular distribution of the sensors was then assessed by immune fluorescent staining of transiently transfected mouse pluripotent neuronal C17.2 cells using an antibody against GFP. Cells were counterstained for calnexin located in the ER and the nuclear envelop and with the dye DAPI binding to the nuclear DNA. This procedure revealed the ubiquitous (i.e. cytosolic and nuclear) distribution of the untargeted GFP_1–10_ sensor, the nuclear localization of the nucGFP_1–10_ sensor that overlapped with the DAPI staining, and the co-localization of the ER-marker calnexin with the erGFP_1–10_ sensor but not with the ommGFP_1–10_ sensor associated to mitochondria^40^ (Fig. 1a). The specificity of α-GFP staining was highlighted by the absence of signal in the surrounding untransfected cells, which were positive only for calnexin and DAPI (Fig. 1a). A subcellular distribution consistent to that observed in C17.2 cells was obtained in human kidney HEK293 cells and in human SH-SY5Y neuroblastoma cells (Supplementary Fig. 1).

**Figure 2:**
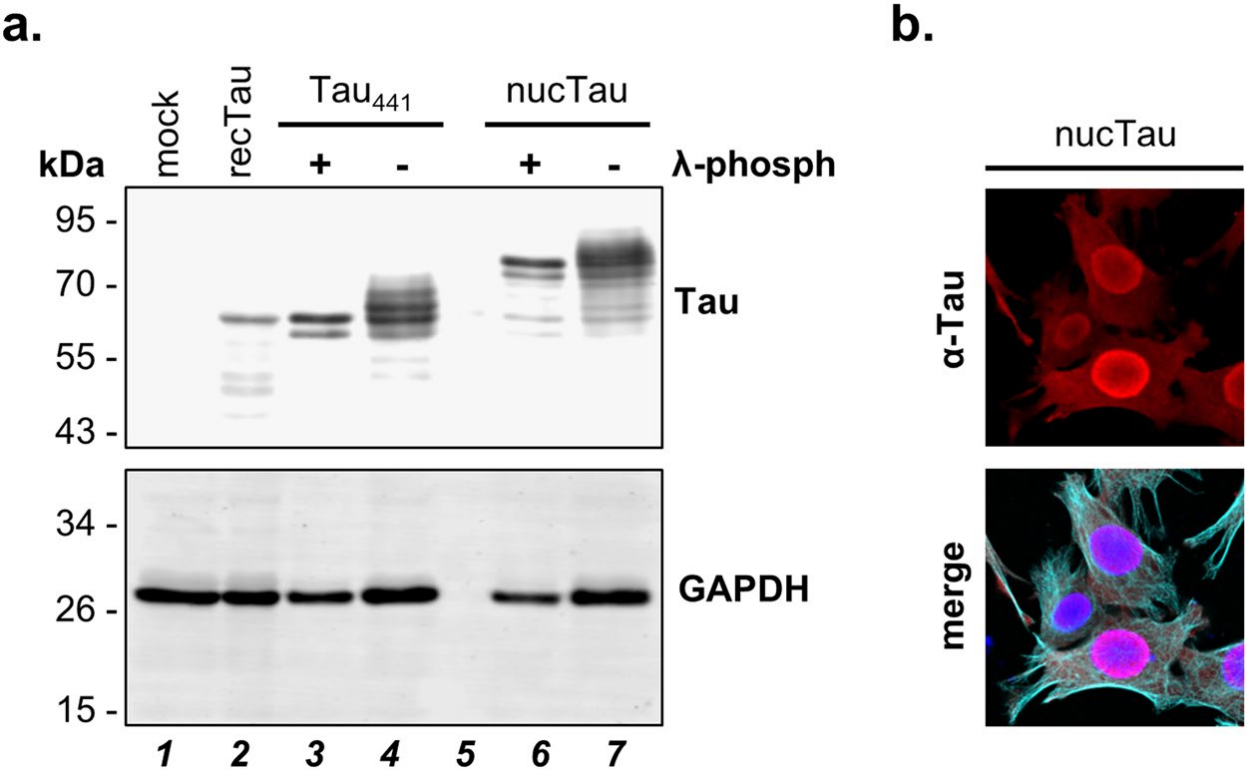
Tau and nuclear targeted Tau are phosphorylated in C17.2 cells. (**a**) Cell extracts (10 µg total protein) obtained from C17.2 cells transiently transfected with Tau (Tau_441_) or nuclear targeted Tau (nucTau) are analysed by western blot with the human-specific Tau13 antibody in the absence or presence of λ-phosphatase treatment (λ-phosph). Bacterial recombinant Tau was mixed with a mock transfected cell lysate (recTau_441_). GAPDH served as a loading control. (**b**) Confocal microscopy images of C17.2 cells demonstrate nuclear targeting of nucTau (Tau13 staining, in red). Cells are counterstained for microtubules (α-tubulin, in cyan) and the nucleus (DAPI, in blue in the merged image).

In order to reconstitute GFP fluorescence, we then co-transfected C17.2 cells with the longest – 441 amino-acid long – isoform of human Tau fused at the amino-terminus with the eleventh β-strand of GFP (S_11_). S_11_ complements the GFP_1–10_ sensor for reconstituting a biofluorescent GFP. Co-location of S_11_-Tau and GFP_1–10_, resulted in strong cytosolic reconstituted GFP-fluorescence (biFC) (Fig. 1b). Here, cells were counter-stained with a human-specific Tau antibody, which revealed accurate co-localization of biFC with the expected cellular distribution of Tau along microtubules. The use of the nucGFP_1–10_ sensor revealed the presence of Tau within the cell nucleus, whereas ommGFP_1–10_ demonstrated mitochondria-associated Tau. Thus, although the main pool of Tau was again detected in the cytosol by the α-Tau antibody, the two targeted sensors visualized minor pools of Tau within the nucleus or bound to mitochondria (Fig. 1b). Microtubule-associated Tau was better visualized with a β-tubulin antibody when the cells were fixed in ice-cold methanol^38^, on the other hand methanol fixation was less suited to reveal the GFP biofluorescent signal and protein localization within the nucleus. No biFC was obtained with the erGFP_1–10_ sensor (Fig. 1b) and for that matter also with a GFP_1–10_ sensor targeted to the mitochondrial matrix (Supplementary Fig. 2)^18^ indicating the absence of detectable Tau protein levels within the secretory pathway or mitochondria. In order to show the functionality of the erGFP_1–10_ sensor, we generated a construct encoding for a Tau carrying at its amino-terminus the signal peptide of influenza hemagglutinin followed by a consensus sequence for amino-glycosylation, whereas the S_11_-peptide was added at the carboxy-terminus (erTau-S_11_). Tau targeted to the lumen of the ER complemented the erGFP_1–10_ sensor and generated a biFC signal (Fig. 1b) that co-localized with the ER-maker calnexin. Additional evidence for the presence of erTau-S_11_ in the secretory pathway was its significantly slower migration on SDS PAGE due to plausible glycosylation (see below Fig. 2b, compare lanes 7 and 4) and robust secretion.

The aim of this study was to assess whether distinct subcellular location of Tau may affect its post-translational modification. So, the next step was to first examine whether C17.2 cells actively modified Tau. For this, we analysed Tau on SDS polyacrylamide gel electrophoresis followed by western blot with the Tau13 monoclonal antibody (Fig. 2a, lane 4; Supplementary Fig. 3). This analysis revealed that human Tau in C17.2 cells displayed a heterogeneous apparent molecular weight, as known for phosphorylated Tau. Consistent with this interpretation, the majority of the Tau forms on the western blot had a larger apparent molecular mass than that of the unmodified recombinant Tau_441_ expressed in bacteria (Fig. 2a, lane 2; Supplementary Fig. 3). Treatment of cell lysates with λ-phosphatase reduced heterogeneity and apparent molecular mass of Tau expressed by C17.2 cells, comparably to that of recombinant Tau (Fig. 2a, lane 3; Supplementary Fig. 3). Similar migration pattern and sensitivity to λ-phosphatase was observed also for endogenous human Tau and for overexpressed Tau_441_ in human SH-SY5Y cells, for overexpressed Tau_441_ in human HEK293 cells and for S_11_-Tau in mouse C17.2 cells (Supplementary Fig. 4). Moreover, Tau protein forced into the nucleus by the same nuclear import signal used for the sensor (nucTau, Fig. 2b) showed multiple complex post-translational modification and sensitivity to λ-phosphatase (Fig. 2a, lanes 6 and 7; Supplementary Fig. 3). Specificity of the human-specific mouse monoclonal Tau13 antibody was emphasized by the absence of immune positive signals in mock-transfected mouse C17.2 cells, despite the presence of endogenous mouse Tau (Fig. 2a, lane 1; Supplementary Fig. 3). Reduced GAPDH signal, used as loading control in this experiment, indicated partial unspecific protein degradation in the cell lysates treated with λ-phosphatase when compared to the untreated control (Fig. 2a, compare lanes 3 and 4 or lanes 6 and 7; Supplementary Fig. 3), which may explain the presence of a putative proteolytic fragment of Tau migrating slightly faster than the main Tau band in the λ-phosphatase-treated lysates (Fig. 2a, lane 3; Supplementary Fig. 3).

### Mass Spectrometry reveals subcellular-specific Tau phosphorylation

**Figure 3:**
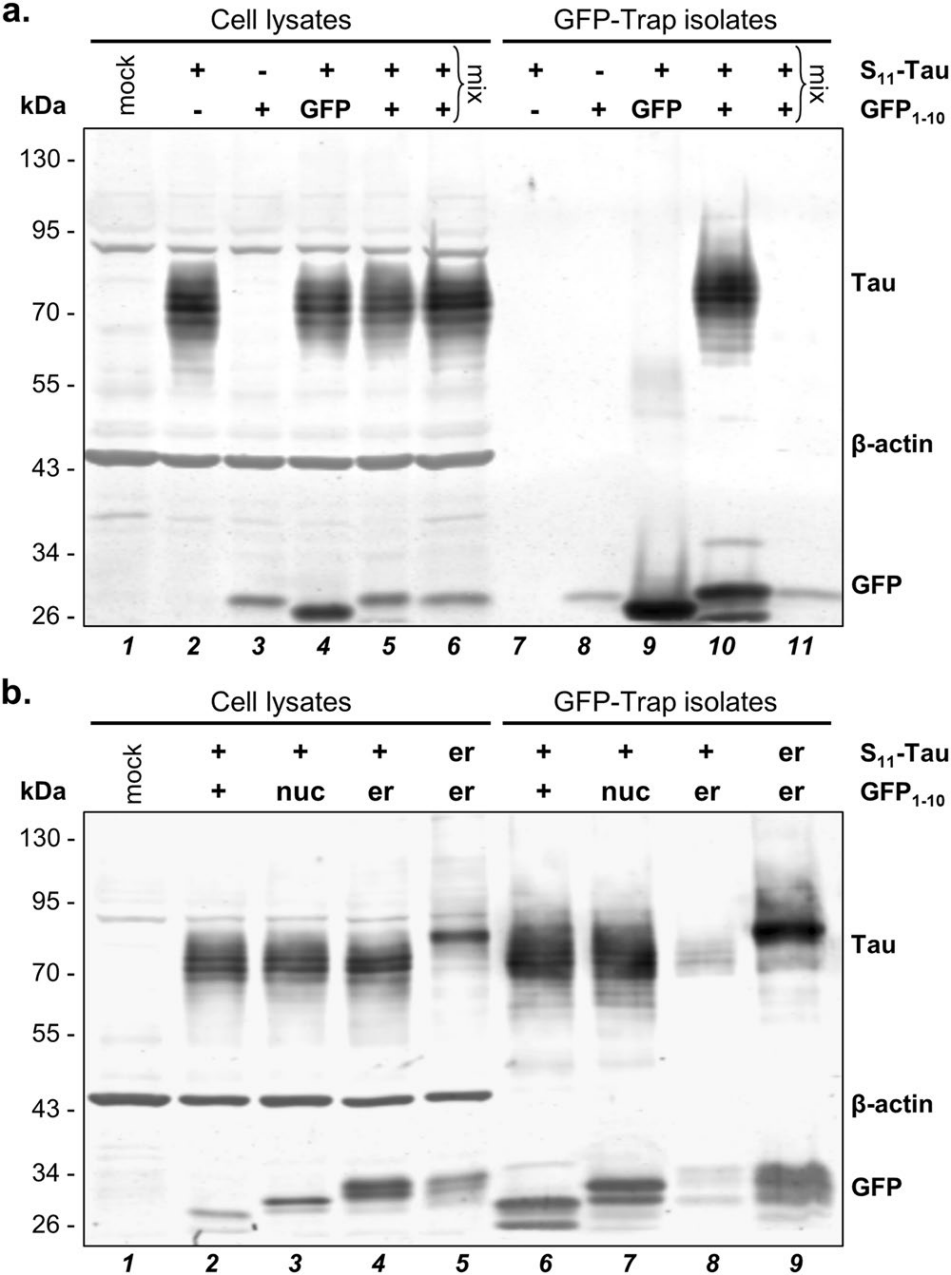
Immune isolation of Tau by mean of the GFP_1–10_ sensors. (**a**) Cell lysates from C17.2 cells transfected as indicated are analysed by western blot before (cell lysates) or after immune isolation on anti-GFP magnetic beads (GFP-Trap isolates). This shows specific isolation of S_11_-Tau when co-transfected with GFP_1–10_, but not in the negative controls, including post-lysis mixing of lysates obtained from cells transfected with either S_11_-Tau or GFP_1–10_ (lanes labelled with “mix”). β-actin is the loading control for the cell lysates, whereas the presence of the sensor is verified with an anti-GFP antibody. (**b**) Cell lysates and GFP-immune isolates obtained from C17.2 cells transiently transfected with S_11_-Tau and the indicated sensors are analysed by western blot as described for (**a**). Molecular weight markers are given on the left of the blots, full blots are shown scanned by dual infrared imaging.

Having established the presence of Tau within the nucleus of C17.2 cells (Fig. 1b) and its post-translational modification by phosphorylation (Fig. 2a), next we optimized conditions for the isolation of S_11_-Tau using the GFP_1–10_ sensors as immune isolation baits, in order to characterize Tau modification depending on its cellular distribution^38^. First, we prepared detergent-free extract obtained from C17.2 cells co-transfected with S_11_-Tau and GFP_1–10_ and demonstrated the presence of the two biFC partners by western blot (Fig. 3a, lane 5). Immune isolation of the GFP-containing biFC complex was performed by single-step affinity purification with anti-GFP V_H_H antibody coupled to magnetic agarose beads (GFP-Trap). This procedure, led to efficient co-purification of S_11_-Tau from extract obtained from cells co-transfected with S_11_-Tau and GFP_1–10_ (Fig. 3a, lane 10). In order to evaluate the specificity of the immune isolation procedure, the negative controls included cell extracts obtained from mock transfected cells (Fig. 3a, lane 1), cells transfected with either one of the two proteins without the respective biFC partner (Fig. 3a, lanes 2, 3, 7, 8), or replacing the GFP_1–10_ sensor with intact GFP (Fig. 3a, lanes 4 and 9). Tau immune reactivity in the GFP-bound fractions was very faint, i.e. at unspecific background levels, in the samples obtained from cells lacking GFP_1–10_ or co-expressing intact GFP instead of GFP_1–10_. Moreover, mixing cell extracts obtained from cells expressing separately S_11_-Tau or GFP_1–10_ before the immune isolation did not result in co-isolation of Tau (Fig. 3a, lanes 6 and 11). This showed lack of post-extraction GFP reconstitution, which reduced the liability of co-isolating Tau protein assembled post-extraction with the GFP_1–10_ sensor when the two binding partners did not co-localized in intact cells. β-actin immune blotting was used as loading control for the unprocessed cell extracts, and its absence in the GFP-immune isolates was a further evidence of the specificity of the enrichment procedure. Furthermore, based on the results obtained with standard immune precipitation with the αGFP rabbit polyclonal ab290 coupled to protein G-agarose beads, it should be noted that GFP-Trap inefficiently isolated unreconstituted GFP_1–10_. Based on these data, we predicted isolation of subcellular Tau pools from cells expressing different targeted GFP_1–10_ sensors by GFP-Trap. This procedure would consent the identification of subcellular location-specific post-translational modifications of Tau, which we did by LC-MS/MS analysis.

**Figure 4:**
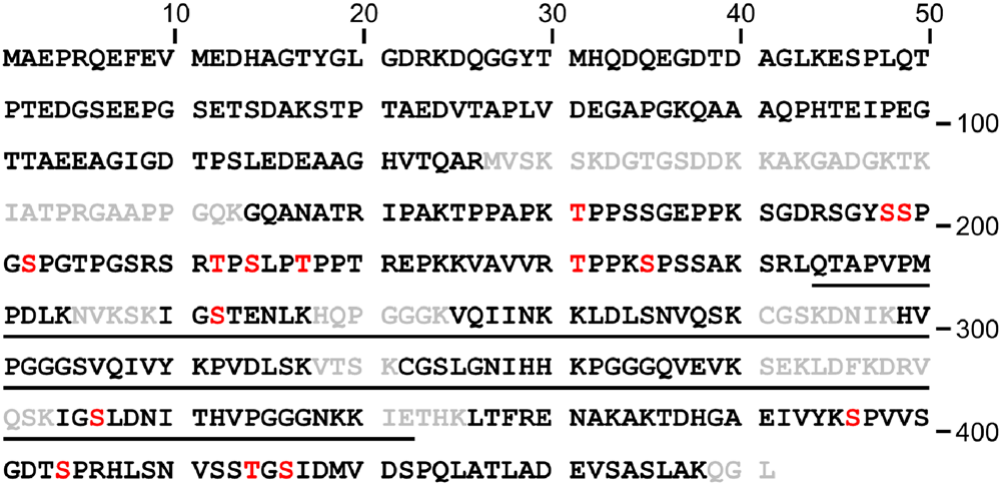
LC-MS/MS reveals Tau phosphorylation. Primary sequence of human Tau_441_ with highlighted sequence coverage (81%; letters in black) and phosphorylation sites (letters in red) revealed by LC-MS/MS. The microtubule binding domain of Tau is underlined.

We then generated scaled-up extracts from C17.2 cells transfected with plasmids encoding S_11_-Tau and the untargeted GFP_1–10_ or the nucGFP_1–10_ sensors (Fig. 3b, lanes 2 and 3) and isolated the biFC complex with GFP-Trap (Fig. 3b, lanes 6 and 7). The specificity of the affinity purification for reconstituted GFP formed before cell extraction was confirmed with the erGFP_1–10_ sensor. Consistent with the biFC data by microscopy (Fig. 1b), Tau-affinity purification by means of erGFP_1–10_ sensor occurred from cells expressing ER-targeted erTau-S_11_ but not when expressing S_11_-Tau (Fig. 3b, lanes 4, 5, 8, 9). The same procedure was also applied to isolate ommGFP_1–10_-bound Tau (Supplementary Fig. 5). Next, GFP-affinity isolated Tau was digested with Lys-C/trypsin and the resulting peptides were analysed by LC-MS/MS. Sequence coverage resulting from this analysis reached 81% of the Tau polypeptide (Fig. 4). Search for post-translationally modified human Tau-derived peptides revealed multiple phospho-threonine and -serine residues (Fig. 4 and Table 1). No other classes of post-translational modifications were detected among those searched, i.e. lysine methylation, acetylation, ubiquitination and nitration. Out of the thirteen phosphopeptides detected, two exhibited double-phosphorylation. The first included two threonines (T_175_ and T_181_; numbering according to the Tau_441_ isoform), and the other a threonine and a serine (T_231_ and S_235_). For all phosphosites detected, the corresponding unmodified residue was also identified, indicating that the sites are partly phosphorylated in C17.2 cells under normal culturing conditions. All samples were analysed twice and we calculated mean peptide intensity from the two measurements, each normalized for the total intensities for all human Tau-derived peptides. We then compared the relative intensities of the phospho-Tau peptides isolated by means of the GFP_1–10_, nucGFP_1–10_ or the ommGFP_1–10_ sensors (Table 1). Significantly increased phosphorylation of peptides embedding T_181_, one of the three S_198/199/202_ (the exact position was not resolved), T_212_ and the double-phosphorylation at T_231_/S_235_ was detected in samples isolated with the nucGFP_1–10_ sensor when compared to GFP_1–10_ or ommGFP_1–10_. A significant increase in the abundance of the phosphopeptide embedding S_404_ was found for nucGFP_1–10_-Tau when compared to GFP_1–10_-Tau, but not for ommGFP_1–10_-Tau, possibly suggesting a differential Tau modification at mitochondria when compared to the cytosol as recently discussed^18^, and also supported by increased pS_356_ and reduced pT_231_/pS_235_ for ommGFP_1–10_-Tau when compared to GFP_1–10_-Tau. For all other phosphosites no differences between the sensors were found. In order to consolidate these results, in a second analysis, we probed biological pentaplicates for the GFP_1–10_ and nucGFP_1–10_ sensors. The overall recovery of peptides in this analysis was lower than the first one, with seven distinct phosphosites detected (Table 2). Here, nucGFP_1–10_-bound Tau was significantly more phosphorylated at T_181_ and T_231_, whereas phosphorylation at the other sites was not significantly different. Overall, the mass spectrometric analysis revealed an increased phosphorylation for Tau isolated with the nucGFP_1–10_ sensor compared to that of the ubiquitously distributed GFP_1–10_ or ommGFP_1–10_, at least at three threonine and two serine residues out of the thirteen phosphosites measured. These findings were unforeseen considering previous work performed by immune staining with the dephosphorylated specific Tau1 antibody^37^. Therefore, we challenged our data by undertaking two independent approaches based on western blot analysis with commercial phospho-specific antibodies.

**Table 1:** First LC-MS/MS mass spectrometric analysis of Tau phosphorylation isolated with different GFP_1–10_ sensors. Tryptic peptides obtained from the indicated GFP_1–10_-Tau immune isolates were analysed twice and the mean peptide intensities, each normalized for the total abundance of all Tau peptides, are given as the relative mean percent for each phosphosite (human Tau441 numbering).

	Ctrl: GFP_1–10_		nucGFP_1–10_		ommGFP_1–10_		nuc vs ctrl		nuc vs omm		omm vs ctrl	
	*n* = 3	*SD*	*n* = 2	*SD*	*n* = 3	*SD*	*p*	*ratio*	*p*	*ratio*	*p*	*ratio*
pT_181_	**2.49%**	0.22%	**3.11%**	0.25%	**2.13%**	0.34%	**0.004**	1.2	**6 × 10** ^**−6**^	1.5	ns	0.9
pT_175_ + pT_181_	**0.05%**	0.03%	**0.04%**	0.02%	**0.01%**	0.01%	ns	0.8	ns	3.3	ns	0.2
pS_198/199/202_	**2.55%**	0.10%	**3.42%**	0.37%	**2.19%**	0.09%	**5** × **10**^**−5**^	1.3	<**10**^**−6**^	1.6	ns	0.9
pT_212_	**0.71%**	0.11%	**1.31%**	0.08%	**0.41%**	0.07%	**0.006**	1.9	**3** × **10**^**−5**^	3.2	ns	0.6
pS_214_	**0.25%**	0.03%	**0.17%**	0.01%	**0.15%**	0.06%	ns	0.7	ns	1.2	ns	0.6
pT_217_	**0.10%**	0.02%	**0.08%**	0.05%	**0.07%**	0.01%	ns	0.8	ns	1.2	ns	0.7
pT_231_	**0.65%**	0.08%	**0.75%**	0.11%	**0.63%**	0.15%	ns	1.2	ns	1.2	ns	1.0
pT_231_ + pS_235_	**3.16%**	0.48%	**3.65%**	0.81%	**1.91%**	0.37%	**0.032**	1.2	<**10**^**−6**^	1.9	<**10**^**−6**^	0.6
pS_262_	**0.01%**	0.01%	**0.02%**	0.01%	**0.04%**	0.03%	ns	2.5	ns	0.5	ns	5.5
pS_356_	**0.16%**	0.02%	**0.17%**	0.13%	**0.57%**	0.55%	ns	1.1	ns	0.3	**0.047**	3.5
pS_396_	**0.02%**	0.01%	**0.01%**	0.01%	**0.01%**	0.01%	ns	0.7	ns	1.0	ns	0.7
pS_404_	**0.28%**	0.02%	**0.78%**	0.19%	**0.53%**	0.02%	**0.027**	2.7	ns	1.5	ns	1.9
pS_416_	**0.19%**	0.04%	**0.11%**	0.03%	**0.07%**	0.06%	ns	0.6	ns	1.5	ns	0.4

2way ANOVA and Tukey’s multiple comparison test (alpha 0.05).

**Table 2:** Second LC-MS/MS mass spectrometric analysis of Tau phosphorylation isolated with GFP_1–10_ or nucGFP_1–10_. Tryptic peptide intensities, each normalized for the total abundance of all Tau peptides, are given as relative percent for each phosphosite (human Tau441 numbering).

	GFP_1–10_		nucGFP_1–10_		*p*	*ratio*
	*mean*	*SD*	*mean*	*SD*		
pT_181_	**0.28%**	0.31%	**0.91%**	0.32%	**0.012**	3.3
pS_198/199/202_	**2.16%**	0.80%	**2.33%**	0.39%	ns	1.1
pT_212_	**0.04%**	0.03%	**0.30%**	0.24%	ns	7.6
pT_231_	**0.50%**	0.26%	**1.16%**	0.43%	**0.009**	2.3
pT_231_ + pS_235_	**0.21%**	0.19%	**0.63%**	0.46%	ns	3.1
pS_262_	**0.05%**	0.03%	**0.11%**	0.07%	ns	2.3
pS_404_	**2.71%**	1.07%	**2.89%**	0.55%	ns	1.1
pS_416_	**0.14%**	0.13%	**0.04%**	0.05%	ns	0.3

2way ANOVA and Bonferroni’s multiple comparison test (n = 5, alpha 0.05).

### Nuclear targeting of Tau modifies its phosphorylation

**Figure 5:**
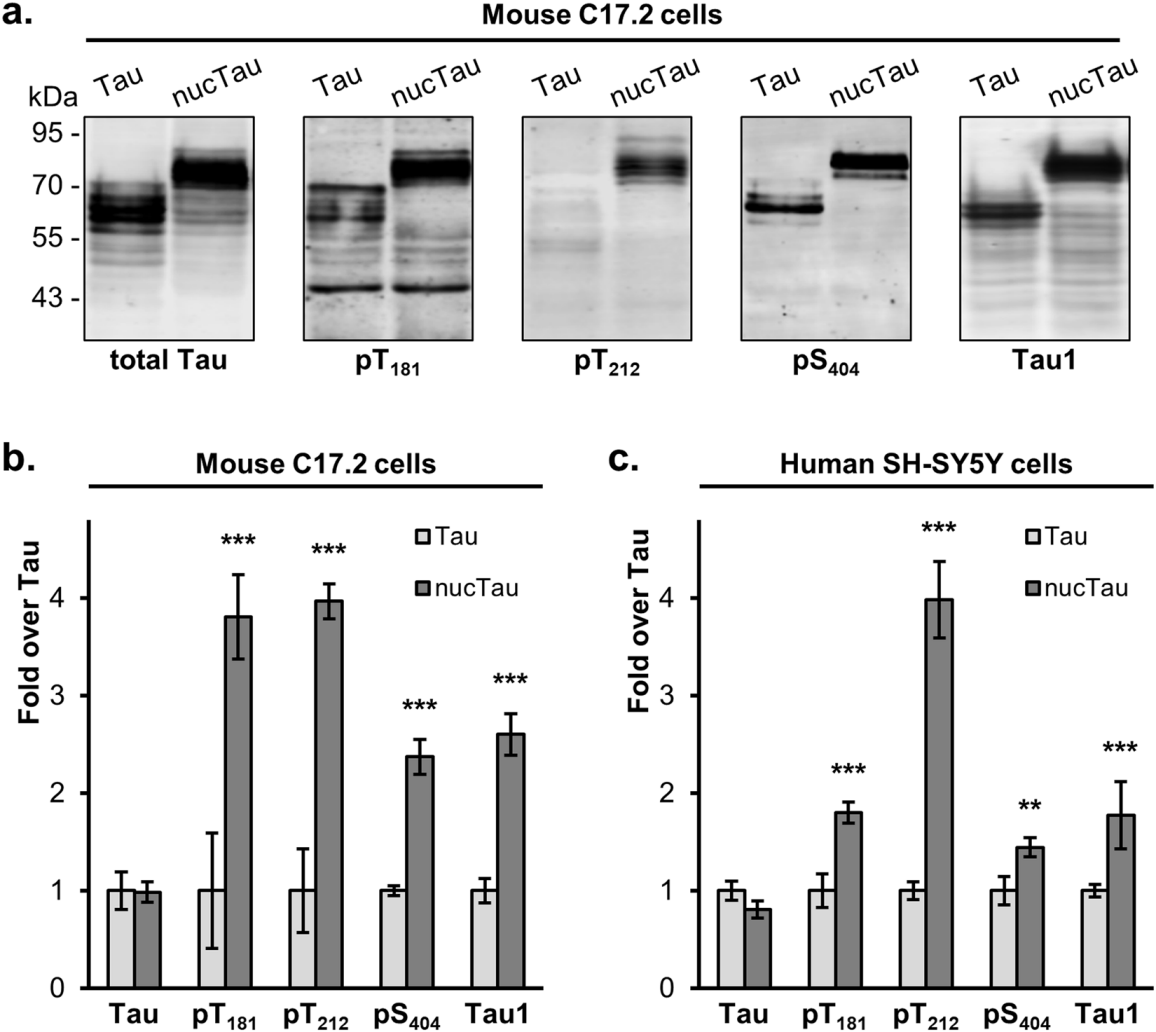
Nuclear targeting of Tau modifies its phosphorylation. (**a**) Cell lysates obtained from C17.2 cells transiently transfected with Tau or nucTau are analysed by western blot with pan-Tau antibody Tau13 or antibodies against the indicated phosphosites or dephosphorylated Ser_195/198/199/202_ (Tau1). Molecular weight markers are shown on the left. (**b** and **c**) Quantification of Tau and nucTau and their modifications in C17.2 (n = 3) or SH-SY5Y (n = 5) cells. Values are mean ± SD. 2way ANOVA and Bonferroni’s multiple comparison test, **p < 0.01, ***p < 0.001.

For the first approach, C17.2 cells were transfected with untagged human Tau_441_ or nuclear targeted nucTau (Fig. 2b). One day later, total cell lysates were analysed by western blot, whereby cell lysates were loaded in a way to obtain similar amounts of Tau (Fig. 5a, Supplementary Fig. 6). Relative phosphorylation, after normalization for Tau measured with the Tau13 pan-antibody, was determined for biological triplicates, and calculated as ratio of the value for nucTau over that of wild-type Tau (Fig. 5b). Phosphorylation was increased for nucTau at the three sites examined, i.e. T_181_ by 3.8 ± 0.4 fold the value of wild-type Tau (p 1 × 10^−9^, 2way ANOVA, alpha 0.05), T_212_ by 4.0 ± 0.2 fold (p 6 × 10^−10^) and S_404_ by 2.4 ± 0.2 fold (p 9 × 10^−5^). Notably, nucTau detection was also increased for the Tau1 antibody with 2.6 ± 0.2 fold (p 1 × 10^−5^), specific for non-phosphorylated Ser_195_/_198_/_199_/_202_^41^. All phosphorylation dependent sites were also increased for nucTau in transiently transfected human SH-SY5Y cells (Fig. 5c, Supplementary Fig. 7): T_181_ by 1.8 ± 0.1 (p 4 × 10^−7^), T_212_ by 4.0 ± 0.4 (p 1 × 10^−15^), S_404_ by 1.5 ± 0.1 (p 0.004) and Tau1 by 1.8 ± 0.3 (p 8 × 10^−7^).

### Analysis of wild-type Tau phosphorylation in a nuclear fraction

**Figure 6:**
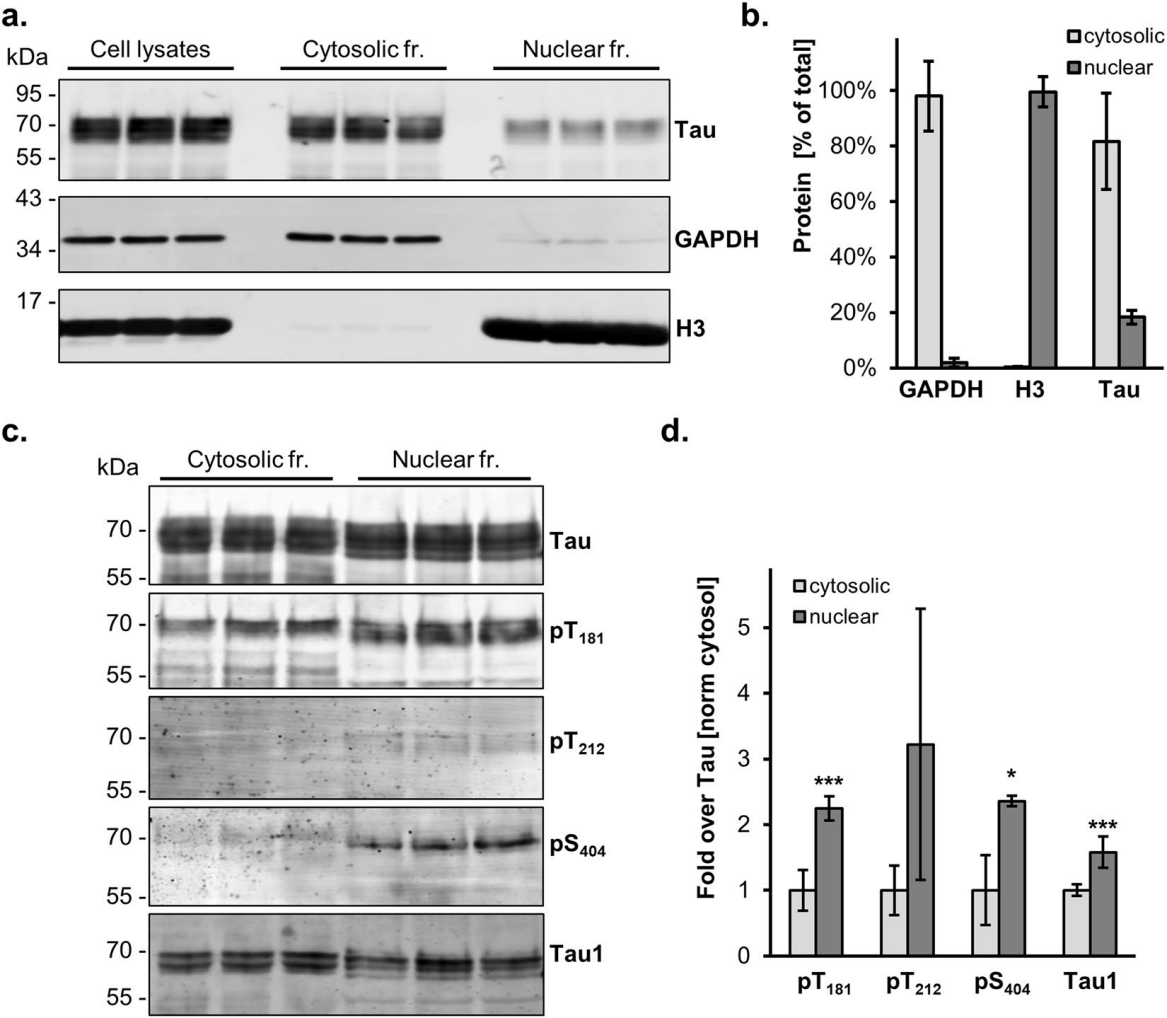
Subcellular fractionation reveals location-specific Tau modification. (**a**) Cell lysates, cytosolic and nuclear fractions obtained from biological triplicates of C17.2 cells induced for human Tau expression for 24 hr are analysed by western blot with antibodies for Tau (Tau13), for the cytosolic GAPDH marker and for the nuclear histone 3 (H3) marker. Molecular weight markers are shown on the left. Full blots are presented in Supplementary Fig. 4 online. (**b**) Quantification is plotted as mean percent ± SD recovered in the two subcellular fractions. (**c**) Cytosolic and TCA-precipated nuclear fractions are analysed by western blot with antibodies for pan-Tau, against the indicated phosphosites or for dephosphorylated Ser_195_/_198_/_199_/_202_ (biological triplicates). (**d**) Quantification of modified Tau normalized for the amount of total Tau detected in each sample. To facilitate the comparison between sites, each modified site is further normalized with the respective values measured in the cytosolic fraction. Values are mean ± SD, *n* = 3. 2way ANOVA and Bonferroni’s multiple comparison test, *p < 0.05, ***p < 0.001.

For the second approach, we took advantage of the availability in the laboratory of a mouse C17.2 cell line with tetracycline-inducible expression of human Tau_441_ in order to isolate enriched cytosolic and nuclear fractions by a differential centrifugation procedure. Purity of the fractions was assessed by immune detection with the cytosolic GAPDH marker and the nuclear H3 marker histone 3 (Fig. 6a, Supplementary Fig. 8). Quantification of the signals obtained from biological triplicates demonstrated for the nuclear fraction a presence of GAPDH reaching 2.0 ± 1.6% for GAPDH, 99.6 ± 5.4% for H3 and 17.3 ± 2.5% for Tau (Fig. 6b). In order to analyse same amounts of Tau for both fractions, the nuclear samples were concentrated by 3-chloroacetic acid (TCA) precipitation before detecting phosphorylated Tau by western blot (Fig. 6c, Supplementary Fig. 9). Again, nuclear Tau displayed increased phosphorylation at T_181_ by 2.2 ± 0.2 fold the value of normal Tau (p 3 × 10^−5^, 2way ANOVA, alpha 0.05.), S_404_ (2.4 ± 0.1 fold; p 0.043) and decreased phosphorylation at the Tau1 epitope (1.6 ± 0.2 fold; p 0.0001) (Fig. 6d). Also T_212_ was found more phosphorylated in the nuclear fraction when compared to the cytosolic fraction, but this did not reach significance (3.2 ± 2.1%; p 0.9), possibly because the detection of pT_212_ in both fractions was only slightly above background determined with samples obtained from untransfected C17.2 cells, resulting in high variation between replicates.

### DNA damage modifies subcellular location and modification of Tau

**Figure 7:**
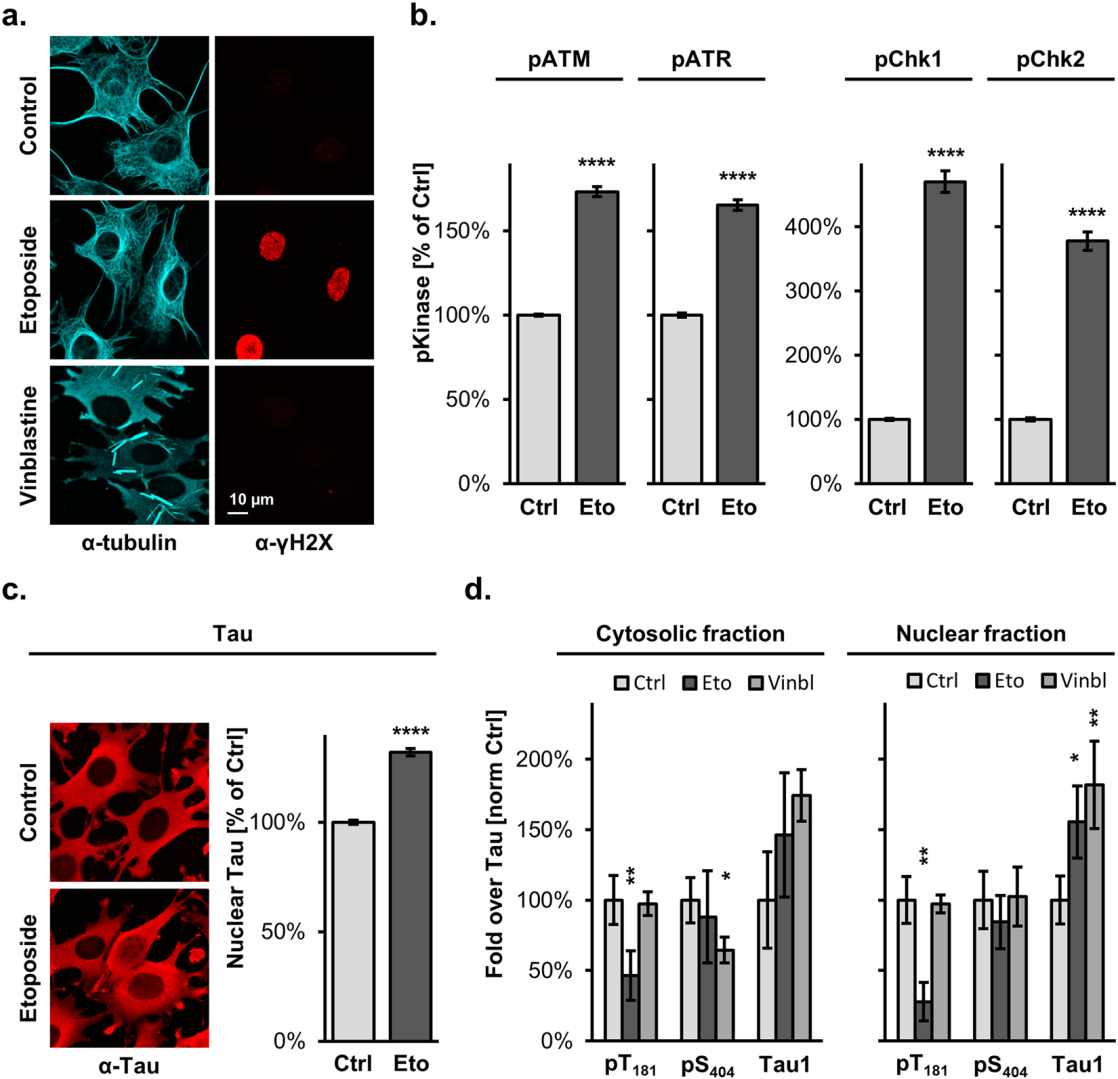
DNA damage induces de-phosphorylation of nuclear Tau. (**a**) The effect of Etoposide and Vinblastine treatment on mouse C17.2 cells is shown by confocal microscopy upon immune staining of PFA-fixed cells with antibodies against the microtubule marker β-tubulin (in cyan) or the DNA damage marker γH2A-X (in red). (**b**) Confocal microscopic quantification of the activated kinases in the nucleus (DAPI mask). Mean percent ± sem relative to the respective controls. 2-tailed unpaired Mann-Whitney test, ****p < 0.0001. (**c**) Confocal microscopy images of C17.2 cells with induced Tau_441_ expression treated in the absence or presence of 60 µM Etoposide for 5 hr and stained for with the human Tau antibody (Tau13). Quantification of immune fluorescent detection of human Tau in DAPI-stained nuclei. Mean percent ± sem relative to the control. 2-tailed unpaired Mann-Whitney test, ****p < 0.0001. (**d**) Cytosolic and nuclear fractions obtained from biological replicates of C17.2 cells with induced Tau expression and treated as indicated are analysed by western blot with antibodies for the two phosphosites pT_181_ and pS_404_ and for the dephosphorylated Tau1 epitope are quantified upon normalization for total Tau. Data represent mean percent ± SD relative to the untreated controls. Non-parametric Kruskal-Wallis test, *p < 0.05, **p < 0.01.

Having established that Tau phosphorylation depends on its subcellular location, we analysed whether compounds expected to affect cellular Tau distribution may influence Tau modification. We focused our attention on treatments known to cause a nuclear Tau translocation following DNA damage, or to interfere with the Tau-microtubule interaction. Microscopic immune-fluorescent analysis of C17.2 cells with induced human Tau expression confirmed that treatment with the topoisomerase II inhibitor Etoposide induced activation of the DNA damage response, visualized with phospho-specific antibodies against γH2A-X (Fig. 7a) or against four nuclear phosphorylated kinases (Fig. 7b). Quantification of the fluorescent signal by confocal microscopy in the DAPI-stained nuclei demonstrated that activation of the early DNA damage response kinases in the presence of Etoposide, when compared to the untreated controls, reached 173 ± 3% for ATM (mean ± sem, n = 218 nuclei, p 10^−15^, 2-tailed unpaired Mann-Whitney test), 165 ± 3% for ATR (n = 212–225, p 10^−15^,), 470 ± 17% for Chk1 (n = 208–214, p 10^−15^,) and 378 ± 14% for Chk2 (n = 200–222, p 10^−15^,). The nuclear signal for Tau in the presence of Etoposide reached 132 ± 2% (n = 857–859, p 10^−15^,) that obtained for control conditions, confirming that the DNA damage caused translocation of Tau to the nuclear compartment (Fig. 7c). Vinblastine caused the collapse of the microtubule network as shown by the presence of brightly fluorescent microtubule fragments in the perinuclear region visualized with a β-tubulin antibody (Fig. 7a) but did not induce nuclear Tau translocation (Supplementary Fig. 10).

In order to evaluate a possible effect of the drug treatments, Tau phosphorylation was analysed by western blot in total cell lysates as well as in cytosolic and nuclear fractions (Fig. 7d). When compared to the control conditions, Etoposide treatment reduced pT_181_ in total lysates to 42 ± 18% (mean ± SD, n = 6, p 0.01, non-parametric Kruskal-Wallis test), in the cytosol to 46 ± 18% (p 0.005) and, notably, even more in the nucleus to 28 ± 14% (p 0.001). An increase of the de-phosphorylated dependent Tau1 epitope to 155 ± 26% (p 0.024) was found in the nuclear fraction, but no significant changes were observed in the cytosolic fraction or in total cell lysates. Etoposide treatment did not affect significantly pS_404_. In contrast, Vinblastine-treated cells displayed reduced pS_404_ to 64 ± 9% (p 0.046, n = 3) in the cytosol and substantially also in total lysates (70 ± 7%, not significant) but not in the nuclear fraction (102 ± 21%). On the other hand, we found increased Tau1 signal in the presence of Vinblastine in total cell lysates to 211 ± 51% (p 0.019, n = 3) and in the nuclear fraction to 182 ± 31% (p 0.024) but this did not reach significance in the cytosolic fraction (174 ± 18%). We also tested the effect of the nuclear export inhibitor Leptomycin b and found a significant increase in the nuclear staining for Tau reaching 144 ± 4% (mean ± sem, n = 154–178 nuclei, p 10^−15^) but this effect on the retention of Tau in the nucleus did not affect any of the phosphosites of Tau analysed in the nuclear fraction, in the cytosolic fraction or in total cell lysates.

In summary, our data show that the phosphorylation of Tau differs depending on its subcellular location and that phosphorylation of nuclear and cytosolic Tau is regulated by different cellular pathways.

## Discussion

With a set of cellular models and analytical methods, we investigated nuclear Tau modification and obtained evidence for different phosphorylation at defined sites in this cellular compartment. First evidence of subcellular location-dependent modification was produced by mass spectrometry of S_11_-Tau isolated with GFP_1–10_ sensors targeted to distinct cellular compartments. In this analysis, phosphorylation of nuclear sensor-bound Tau, when compared to Tau captured with the untargeted sensor, was increased at five out of thirteen Tau phosphopeptides detected by mass spectrometry, namely those including T_181_, S_198/199/202_, T_212_, S_404_ and the double phosphorylated T_231_/S_235_. Reinforcing these data, these residues, with the exception of S_404_, were also more phosphorylated for the nuclear sensor when compared to Tau isolated by means of the sensor targeted to the outer mitochondrial membrane. On the other hand, association to mitochondria reduced pT_231_/pS_235_ but increased pS_356_, confirming the possibility of a mitochondria-localized pool of Tau with unique functions^18^. Previously published work claimed reduced nuclear Tau phosphorylation by immune staining with the dephosphorylated Ser_195/198/199/202_-specific antibody Tau1^37^. Data from our laboratory indicate that the analysis of (de)phosphorylation of endogenous Tau by immune staining of cultured cells needs confirmation with a proper negative control. In fact, using Tau1 and some phospho-Tau antibodies used in this study on human SH-SY5Y cells, wild-type or with destroyed *MAPT* gene, resulted in a nucleus-localized staining that was not dependent on the presence or the absence of endogenous or overexpressed Tau. Nevertheless, because of this and the fact that a second mass spectrometric analysis showed increased nuclear Tau phosphorylation only at residues T_181_ and T_231_/S_235_, we continued our study with two different cellular systems. First, we studied the effect of forced nuclear targeting of Tau (nucTau) by western blot with phospho-specific antibodies. Quantifiable signals were obtained for pT_181_, pT_212_, pS_404_ and for Tau1 both in transfected mouse C17.2 and human SH-SY5Y cells. For both cell lines, increased immune detection at the same four epitopes was found for nucTau. Since the utilization of the nuclear targeted sensor and of nucTau may influence normal cellular Tau distribution, we then investigated Tau modification in isolated nuclei of C17.2 cells expressing human wild-type Tau. This confirmed the presence of Tau in the nucleus at levels higher than those of the potential cytosolic contaminant GAPDH. When compared to the cytosolic fraction, also Tau present in the nuclear fraction displayed increased detection of three out of four epitopes analysed. Our data describe the phosphorylation status of nuclear Tau in proliferative pluripotent neuronal C17.2 and neuroblastoma SY5Y cells and this may not apply in differentiated cells such as neurons.

The presence of Tau in the nucleus was documented previously *in vitro*^23–27^ and *in vivo*^28^ where a diffuse distribution in the nucleoplasm or association to nucleoli in its dephosphorylated, Tau1 antibody-positive form was reported^24,26,27,42^. Within the nucleus, Tau interacts with RNA and DNA^29–32^ and appears to protect the neuron from age-related insults. *In vitro*, Tau raises the melting temperature of DNA^43^. Tau binds and bends DNA when its proline-rich and microtubule-binding domains associate to the AT-rich minor DNA groove independently of the nucleotide sequence^33,36,44^. These data suggest that Tau may shuttle between cytoplasm and nucleus, as canonical heat-shock (HS) proteins do when they induce the HS response^45,46^. Indeed, rescue from HS-induced DNA damage is observed when nuclear-targeted Tau is expressed in Tau-deficient cells or mouse brain^37,47^. Linking the role of Tau in DNA metabolism with disease, cells expressing FTDP-17 mutations display more chromosomal defects that those expressing normal Tau^48^. Here, we show that treatment with the DNA damaging drug Etoposide also causes increased nuclear location of Tau, whereby this is accompanied by decreased phosphorylation at T_181_ and at the Tau1 epitope in the nuclear fraction, without affecting pS_404_. Decreased phosphorylation of Tau in the presence of Etoposide is remarkable in view of the many kinases that are activated during the DNA damage response. Considering the role of phosphorylation in regulating protein-protein interaction and the scaffold nature of Tau as protein linking different binding partner, it is tempting to consider that our data underlie a possible mechanism regulating different functions of Tau. In this regards de-phosphorylation of Tau during the DNA damage response at residue T_181_ and at the Tau1 epitope, i.e. within the proline-reach domain that has been implicated in dephosphorylated Tau-DNA association^44^, may offer a molecular mechanism for the DNA protective role of Tau. As a matter of fact, pT_181_ is the epitope commonly used to show increased phospho-Tau as a cerebrospinal fluid biomarker for an ongoing neurodegenerative process. Our data may indicate that a neurodegeneration-linked increase in pT_181_ could weaken the DNA protecting role of Tau. Interestingly, treatment of the cells with Vinblastine did not increased nuclear Tau localization and did not change pT_181_, but reduced phosphorylation of S_404_ in the cytoplasm but not in the nucleus, suggesting that the carboxy-terminal tail of Tau may have a predominant cytoplasmic function. Increased nuclear Tau retention in the presence of Leptomycin did not affect phosphorylation of Tau at the sites analysed in this study. Notably, increased nuclear Tau was found for the 1N4R isoform of Tau^49^, whereas a pathological form of Tau was shown to interfere with nuclear import and export^50^, indicating that additional mechanisms regulating nuclear Tau translocation and modification exist.

Tau alters the integrity of cytoplasmic and nuclear RNA^47^ and may regulate RNA metabolism when present in stress granules, which protect RNA from degradation during cellular stress^51^. Nuclear localization and a role of Tau in DNA and RNA homeostasis are reminiscent to TDP-43 and FUS, two other proteins involved in frontotemporal dementia (FTD)^52,53^. These are RNA binding proteins that play key roles in transcription, mRNA splicing, stability and transport. Their subcellular location is affected by autosomal dominant FTD mutations^52,53^. It is tempting to consider a common pathway for Tau/TDP/FUS-linked FTD. Whilst FUS is clearly involved in the DNA damage response^54–56^, a role of TDP-43 and Tau appears less evident. Further knowledge on nuclear Tau may provide clues in the understanding of FTD involving TDP-43 and FUS proteins. Due to the wide distribution in the CNS and periphery, Tau is potentially a global player for genome surveillance, in particular against DNA insults accumulating as a function of aging.

## Materials and Methods

### Expression plasmids

cDNAs were inserted in the constitutive expression vector pcDNA3 or in pcDNA5 for inducible expression. For nucGFP_1–10_, the GFP_1–10_ plasmid^38^ was modified at the amino-terminus with a nuclear targeting sequence consisting of an artificial peptide carrying three tandem sequence derived from the SV40 large T antigen (MDIDPKKKRKVDPKKKRKVDPKKKRKVD)^39^. To insert this sequence, we used as template GFP_1–10_ for a PCR with the three overlapping forward primers TC**GGATCC**ATGGACCCGAAGAAGAAACGCAAGGTCGATCCGAAGAAG, CAAGGTCGATCCGAAGAAGAAGCGGAAGGTCGATCCAAAGAAAAAAAGG and CGATCCAAAGAAA AAAAGGAAGGTGTCCAAAGGAGAAGAACTG and the reverse primer TTCA**CTCGAG**CTATGTTCCTTTTTCATTTGG. This inserted also the restriction sites BamHI and XhoI (in bold) for subcloning the amplified fragment in pcDNA3, whereby the initial methionine of GFP was eliminated. The signal peptide at the amino-terminus of erGFP_1–10_ (MLLSVPLLLGLLGLAVA) was that of human calreticulin and was obtained with the forward primer CACGCA**GGAATTC**ATGCTGCTATCCGTGCCGCTGCTGCTCGGCCTCCTCGGCCTGGCCGTCGCCTCCAAAGGAGAAGAACTG (the initial methionine of GFP was eliminated) and the reverse primer ACTTCTCA**CTCGAG**TTACAGCTCGTCCTTTGTTCCTTTTTCATTTGGATC, which also inserted a KDEL sequence for retention in the ER before the stop codon of GFP_1–10_^40^.

Tau expression plasmids encoded the 441 amino acid-splice variant 2N4R of human Tau. The plasmid encoding for untagged Tau, S_11_-Tau, and Tau-S_11_ were generated as described^38^. The nuclear targeting sequence of nucTau was the same used for nucGFP_1–10_ and obtained with forward primer ATAT**AAGCTT**ACCATGGATATCGACCCGAAG and reverse primer TTAA**GGATCC**ATCCACCTTCCTTTTTTTCTTTGG. The amplified fragment was then inserted before the Tau cDNA at the restriction sites HindIII and BamHI (in bold) to obtain the desired polypeptide with the nuclear targeting sequence followed by a short linker (GST) and the sequence of human Tau including the initial methionine. The plasmid encoding for erTau-S_11_, was generated using as template Tau-S_11_, and the primers TTC**GGATCC**ATGAAGACCATCATTGCTTTGAGCTACATTTTCTGTCT GGCTCTCGGCCAAGACCTTCCAGGAAATGACAACAGCACAGCAGCTGAGCCCCGCCAGG and TTCA**CTCGAG**TCATGTGATGCCGGCGGCGTTC followed by subcloning with BamHI and XhoI (in bold) in pcDNA3. The inserted sequence corresponded to MKTIIALSYIFCLALGQDLPGNDNSTA, which covered the signal peptide and the amino-glycosylation consensus sequence (underlined) of influenza A hemagglutinin without the initial methionine of Tau.

### Cell culturing, cell lines and plasmid transfections

Mouse multipotent neural progenitor C17.2 cells and human neuroblastoma SH-SY5Y cells were cultured as described previuosly^38^. Inducible C17.2 cell lines were generated with the Flp-In T-Rex tetracycline-inducible cell system according to the manufacturer instructions (Invitrogen, K650001) and were selected and maintained in the presence of 150 µg/mL Hygromycin B and 15 µg/ml Blasticidin S. Protein expression was induced by one day-incubation with 30 ng/mL Tetracycline, a treatment time and dose that were sufficient to reach steady-state levels of expression. Plasmid transfections were performed with Lipofectamine 3000 (Invitrogen, L-3000-008) as described^38^.

### Drug treatments

During the last 5 h of tetracycline incubation, C17.2 cells with inducible Tau expression were treated with 60 µM Etoposide (Abcam, ab120227; 100 mM stock in DMSO), 3 µM Vinblastine (Sigma-Aldrich, V1377; 11 mM stock in DMSO), or 60 nM Leptomycin B (Sigma-Aldrich, L2913; 10.3 µM in 70% ethanol). At the end of the drug treatment, cells were fixed and processed for fluorescence microscopy. For the immune fluorescence quantification of nuclear Tau and activated kinases, a DAPI mask on ImageJ was used.

### Fluorescence microscopy

For immune fluorescence microscopy, cells were routinely grown on 8-well slides (Ibidi, 80826-IBI) coated with poly-D-lysine. One day after plasmid transfection, cells were fixed either in paraformaldehyde (PFA) or in methanol. For PFA, a 4% solution in PBS pre-warmed at 37 °C was added 1:1 (V:V) to the culture medium for 10 min at 37 °C, followed by a second 5 min fixation in 4% PFA at room temperature, 5 min quenching in 100 μM glycine and three washes with PBS. For methanol, the culture medium was replaced with methanol chilled at −20 °C followed by 10 min incubation at −20 °C. Then, the methanol solution was removed by aspiration and the fixed cell layer gently washed three times with PBS. For the immune staining procedure, all steps were performed at room temperature. The fixed cells were blocked with 5% normal goat serum, 0.3% Triton X-100 in PBS for 1 h followed by PBS washes. The antibodies were diluted in a working solution composed of 0.5% normal goat serum, 0.3% Triton X-100 in PBS. Primary antibodies, usually incubated for 1 h at 37 °C, were specific for human Tau (Tau13, Santa Cruz, sc-21796, used at 1 μg/mL), GFP (Proteintech Europe, 66002–1-Ig, 7 μg/mL), calnexin (kind gift of Prof. Maurizio Molinari, IRB, Bellinzona, Switzerland, diluted 1:1,000), α-tubulin (Abcam, ab1825, 0.5 μg/mL or Cell Signaling, DM1A, diluted 1:500), pS_129_-H2A.X (Santa Cruz, sc-517348, 0.5 μg/mL), pATM (Cell Signaling, S1981, diluted 1:500), pChk1 (Cell Signaling, S345, diluted 1:500), pChk2 (Cell Signaling, T68, diluted 1:500), pATR (Cell Signaling, S428, diluted 1:500). Secondary antibodies (2 μg/mL for 1 h at room temperature in the dark) were anti-mouse IgG (Thermo Fisher Scientific, Alexa594, A-11032, or Alexa 488, A-11001) or anti-rabbit IgG (Thermo Fisher Scientific, Alexa594, A-11037, or Alexa488 A-11034). Nuclei were counterstained with DAPI (0.5 μg/mL). After the staining, cells were kept in 0.05% sodium azide in PBS in the fridge before analysis with a confocal microscope (Nikon C2). Images were taken with a line by line scan using a sequence of excitation with the 405 nm laser and emission filter 464/40–700/100 nm, 488 nm laser and emission filter 525/50 nm, and 561 nm laser and 561/LP nm emission filter.

### Cell lysis and western blotting

Cells were rinsed once with PBS and collected by scraping and low speed centrifugation. Cell lysates were prepared in 100 μL ice-cold RIPA supplemented with inhibitor cocktails for proteases (Sigma-Aldrich, S8820) and phosphatases (Sigma-Aldrich, 04906845001). All samples were then maintained on ice or at 4 °C. Cell lysates were shaken for 30 min at 1,400 rpm (Eppendorf, Thermomixer 5355) and RIPA extracts collected after centrifugation for 10 min at 20,000 g. Total protein concentration was determined by Bradford (Pierce, BCA Protein Assay Kit 23227). For phosphatase treatment, RIPA extracts (10 μg protein), prepared without phosphatase inhibitors, were incubated in the absence or presence of 20 U/μL Lambda PP (BioLabs, P0753S) for 1 h at 30 °C as described by the supplier. Total cell lysates were prepared by direct solubilisation of cell pellets in 100 μL sample buffer: 1.5% SDS, 8.3% glycerol, 0.005% Bromophenol blue, 1.6% β-mercaptoethanol and 62.5 mM Tris-HCl pH 6.8.

SDS-polyacrylamide gel electrophoresis was performed on hand-casted 10% gels at constant 90–110 V (Bio-Rad, Mini-PROTEAN 1658033FC). Protein was transferred on PVDF membranes (Merck-Millipore, Immobilon-FL IPFL00010) using a Trans-Blot Turbo Transfer System (Bio-Rad, 1704150). After the transfer, the membranes were incubated in blocking buffer (Licor Biosciences Odyssey, 927–50000) for 1 h at room temperature. Bound primary antibodies were revealed by the two secondary antibodies anti-mouse IgG coupled to IRDye RD 680 or anti-rabbit IgG coupled to IRDye 800CW (Licor Biosciences, 926–68070 and 926–32211) on a dual infrared imaging scanner (Licor Biosciences, Odyssey CLx 9140) and quantified with the software provided (Licor Biosciences, Image Studio V5.0.21, 9140–500). Antibodies used for western blot were Tau13 (0.2 μg/mL), Tau1 (Merck-Millipore, MAB-3420, 0.5 μg/mL), phospho-Tau T_181_ (AT270, Invitrogen, MN1050, 0.2 μg/mL), T_212_ (Abcam, ab4842, 1:1,000), S_404_ (Invitrogen, 44–758 G, 1:1,000), GAPDH (Abcam, ab181602, 1:2,500), histone 3 (Abcam, ab176842, 1:10,000), β-actin (Sigma-Aldrich, A1978, 0.084 μg/mL), and GFP (Abcam, ab5449, 1:5,000).

### Samples preparation for LC-MS/MS

Detergent-free extracts were prepared in PBS supplemented with protease and phosphatase inhibitors by three freeze/thaw cycles in liquid nitrogen followed by centrifugation for 10 min at 20,000 g. Immune isolation of complemented GFP was performed on magnetic beads. For this, cell extracts obtained by pooling biological triplicate (at least 600 μg total protein) were brought to 500 μL with PBS, supplemented with 5 μL of 50% magnetic bead slurry GFP-Trap (ChromoTek, gtma-20) and incubated on an orbital rotator (Labnet, 096621) for 2 h at 4 °C. With the aid of a magnet, the beads were washed twice with PBS and once with water. A small portion of the beads was eluted with sample buffer to analyse the immune isolates by western blot.

Proteolytic peptides were obtained by double digestion. For this, GFP-trap bead-bound immune isolates were resuspended in 50 µL 50 mM NH_4_HCO_3_ and 8 M urea and reduced in 2 µM dithiotreitol for 1 h at 37 °C with shaking at 800 rpm. Proteins were then alkylated in 4.5 mM iodoacetamide for 45 min in the dark at room temperature. Sequential digestion was performed for 3 h with Lys-C (WAKO, 125–05061; 20 ng/µL), and then overnight with trypsin (Promega, V5113; 2.5 ng/µL) by adding 144 µL 50 mM NH_4_HCO_3_. The digestion was stopped by acidification to pH < 3 with 50% formic acid. The peptide mixtures were loaded on C18 spin tips (Pierce, 84850), and desalted twice with 0.1% formic acid by centrifugation for 1 min at 100 g. Peptides were eluted twice with 80% acetonitrile, lyophilized (Eppendorf, Concentrator plus 5305), solubilized in 0.1% formic acid and analysed by mass spectrometry.

### LC-MS/MS

Samples were measured on a nanoLC (Thermo Fisher, EASY-nLC 1000) coupled to a mass spectrometer (Thermo Fisher, Q Exactive Plus). Peptides were separated on a 40 cm × 0.75 μm column packed in-house with reversed-phase resin (ReproSil-Pur C18- AQ 1.9 μm, Dr. Maisch). Peptides were eluted for 60 min using a segmented linear 5–40% gradient (solvent A: 0.1% formic acid; solvent B: 99.9% acetonitrile, 0.1% formic acid) at 300 nL/min. Survey full-scan mass spectra were acquired with mass range 350–1,500 m/z, at a resolution of 70,000 at 200 m/z and the 20 most intense ions above an intensity of 3.6e4 were sequentially isolated, fragmented (HCD at 25 NCE) and measured at a resolution of 17,500 at 200 m/z. Peptides with a charge of + 1 or with unassigned charge state were excluded from fragmentation for MS2, and a dynamic exclusion of 30 s was applied. Ions were accumulated to a target value of 3e6 for MS1 and of 1e5 for MS2.

In Sequest HT, mass spectra were searched against a mouse database (Uniprot, downloaded July 8th, 2016; supplemented with sequences of human Tau). Carbamidomethylation of cysteine was set as a fixed modification and oxidation of methionine and phosphorylation of serine, threonine and tyrosine were allowed as a variable modification. Precursor mass tolerance was 10 ppm and fragment mass tolerance 0.02 Da. Trypsin was set as enzyme allowing for maximally two missed cleavages. Percolator was used to filter the results to 1% FDR at peptide level and phosphoRS 3.0 for phosphorylation-site localization. Skyline 3 was used to extract the monoisotopic and the first and second isotope precursor intensity traces of all identified Tau peptides.

### Cytosolic and nuclear fractionation

The protocol was derived from an online method (Abcam). All samples were maintained on ice and all centrifugations were performed at 4 °C. C17.2 cells were washed once with PBS and scraped in hypotonic buffer: 250 mM sucrose, 10 mM KCl, 2 mM CaCl_2_, 1.5 mM MgCl_2_, 20 mM HEPES pH 7.4, supplemented with protease and phosphatase inhibitors. Cells were broken by passing the cell suspension four times through a 25-gauge needle. An aliquot of this sample was collected and labelled as “total”. The cytosolic fraction was obtained as supernatant of a sequential centrifugation for 5 min at 1,000 g and for 10 min at 20,000 g. For the nuclear fraction, the pellet from the first centrifugation was washed twice by sequential resuspension in hypotonic buffer, ten-time passage through a 25-gauge needle and centrifugation for 10 min at 1,000 g. The final pellet was resuspended in RIPA supplemented with 1.25 mM MgCl_2_, protease and phosphatase inhibitor, treated with Benzonase nuclease (Merck-Millipore, 70746–4, 0.56 U/µL) for 15 min at 37 °C and collected as the supernatant of a final centrifugation for 10 min at 20,000 g.

In order to concentrate the nuclear fraction, this was mixed with 1 volume of 20% TCA, incubated for 30 min on ice and centrifuged 20 min at 20,000 g. The pellet was washed with −20 °C cold 100% acetone, followed by centrifugation for 20 min at 20,000 g. After supernatant removal, residual acetone was evaporated at room temperature and the samples resuspended in SDS PAGE sample buffer and neutralization with 1 M Tris-HCl pH 10.8

### Statistical analysis

Statistical analysis was performed with the aid of GraphPad Prism version 7.02 as indicated in the results section and figure legends.

## Electronic supplementary material

Supplementary figures
