# Supplementary material for: Phosphorylation of nuclear Tau is modulated by distinct cellular pathways
Source: Sci Rep. 2018 Dec 7;8:17702. doi: 10.1038/s41598-018-36374-4 (PMC6286375; doi:10.1038/s41598-018-36374-4)
Supplement: Supplementary file 1 — Supplementary figures [file 41598_2018_36374_MOESM1_ESM.docx]

**SUPPLEMENTARY FIGURES**

**Phosphorylation of nuclear Tau is modulated by distinct cellular pathways**

Giorgio Ulrich^1^, Agnese Salvadè^1^, Paul Boersema^3^, Tito Calì^2^, Chiara Foglieni^1^, Martina Sola^1^, Paola Picotti^3^, Stéphanie Papin^1^, Paolo Paganetti^1,*^

Affiliations:

1. Laboratory for Biomedical Neurosciences, Neurocenter of Southern Switzerland, Ente Cantonale Ospedaliero, Torricella-Taverne, Switzerland
2. Institute of Molecular Systems Biology, Department of Biology, ETHZ, Zurich
3. Department of Biomedical Sciences and Padova Neuroscience Center, University of Padova, Padova, Italy

* Corresponding author:

Dr. Paolo Paganetti, Head Laboratory for Biomedical Neurosciences, Group Leader Neurodegeneration Research, c/o SIRM, Via ai Söi 24, CH-6807 Torricella-Taverne, Switzerland. Tel +41 91 8117250. paolo.paganetti@eoc.ch

**
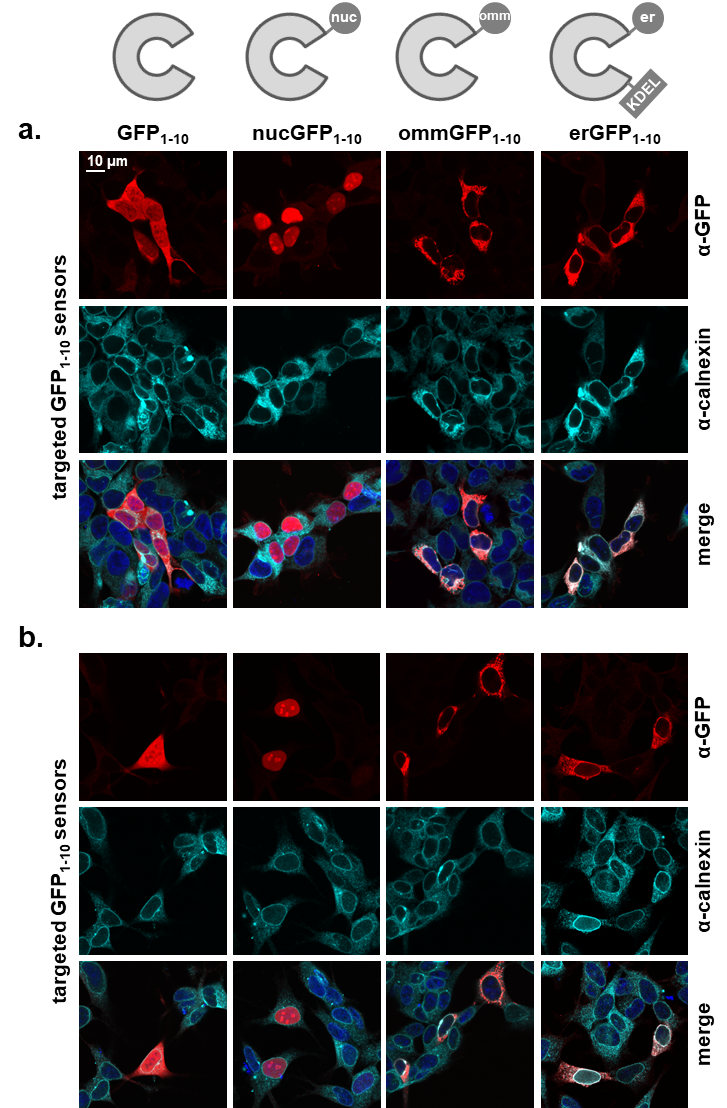
**

**Supplementary Figure 1**

The subcellular distribution of the indicated GFP_1-10_ sensors in transiently transfected human HEK-293 (**a**) or SH-SY5Y (**b**) cells is shown by confocal microscopy upon immune staining of PFA-fixed cells with an anti-GFP antibody (upper row, in red). The cells are counter-stained with the ER-marker calnexin (middle row, in cyan) and the nuclear stain DAPI (shown in the merged images, bottom row, in blue).


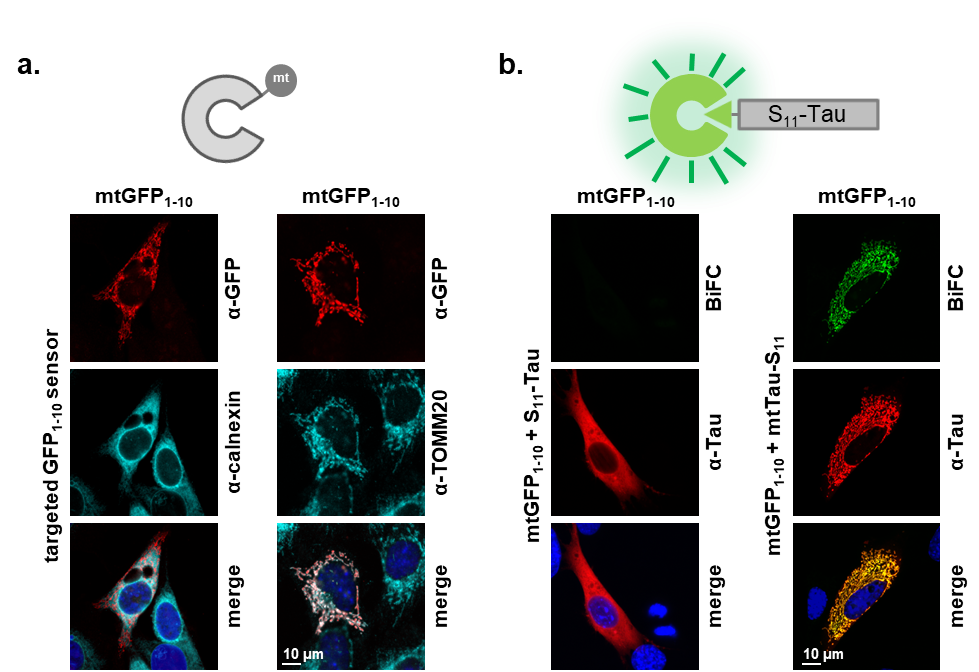


**Supplementary Figure 2**

(**a**) The subcellular distribution of the mitochondrial matrix-targeted mtGFP_1-10_ sensor in transiently transfected C17.2 cells is shown by confocal microscopy upon immune staining of PFA-fixed cells with an anti-GFP antibody (upper row). The cells are counter-stained with the ER-marker calnexin (left column) or the mitochondrial marker TOMM-20 (right column) and the nuclear stain DAPI (merged images). (**b**) Confocal microscope images of cells co-transfected with the mtGFP_1-10_ sensor and S_11_-Tau (left column) or mitochondrial matrix-targeted mtTau-S_11_ (right column). BiFC biofluorescence (upper row) is detected only when both proteins are targeted to the mitochondrial matrix. The cells are counter-stained for human Tau with the Tau13 pan-antibody (middle row) and DAPI (merged images).


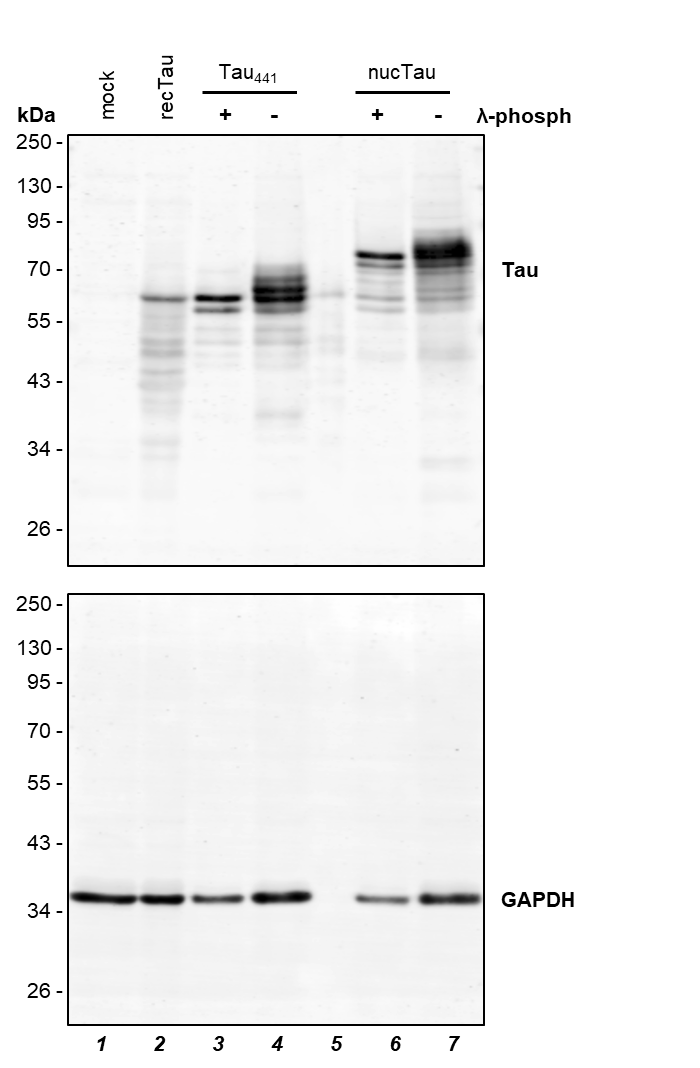


**Supplementary Figure 3**

Full western blots of the two panels shown in Fig. 2a blotted with Tau13 and anti-GAPDH antibodies and scanned by dual infrared fluorescence imaging. Recombinant Tau_441_ is mixed with lysates of mock transfected cells. All samples are derived from the same experiment and processed in parallel.


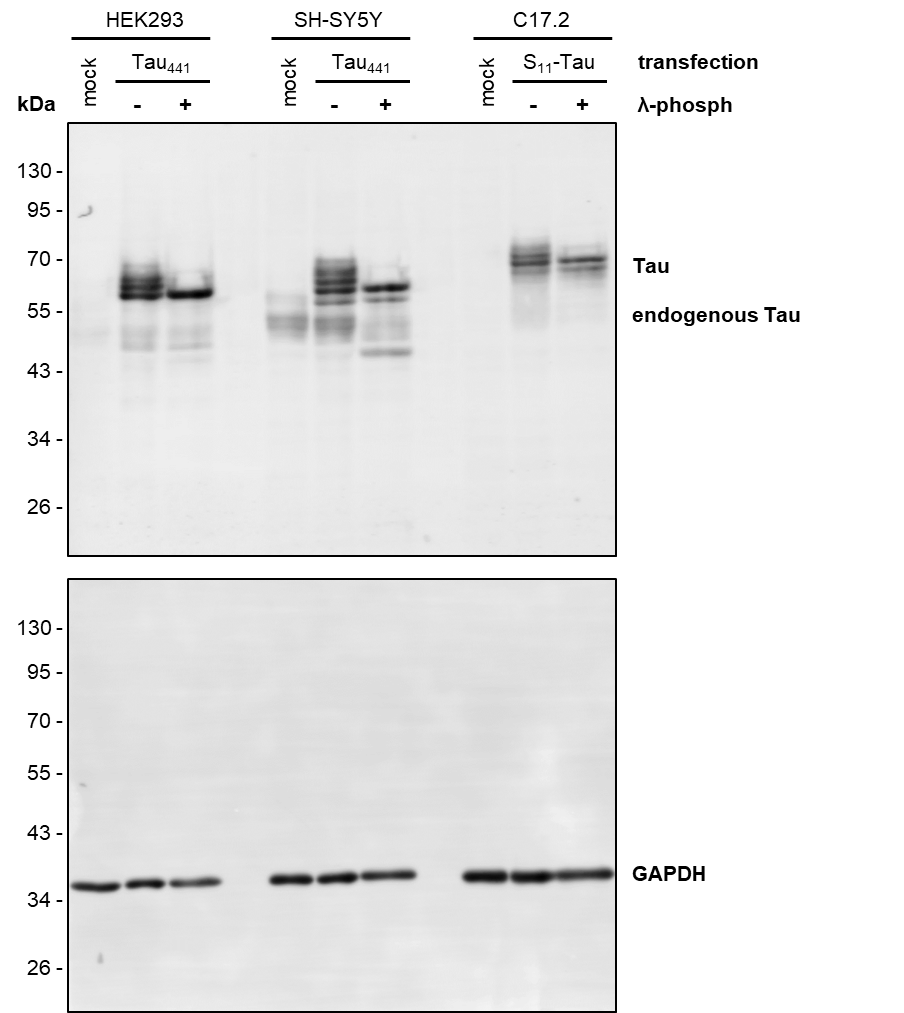


**Supplementary Figure 4**

Full western blots of the of cell lysates obtained from the indicated cell lines treated in the absence or presence of λ-phosphatase, blotted with Tau13 and anti-GAPDH antibodies and scanned by dual infrared fluorescence imaging. All samples are derived from the same experiment and processed in parallel.


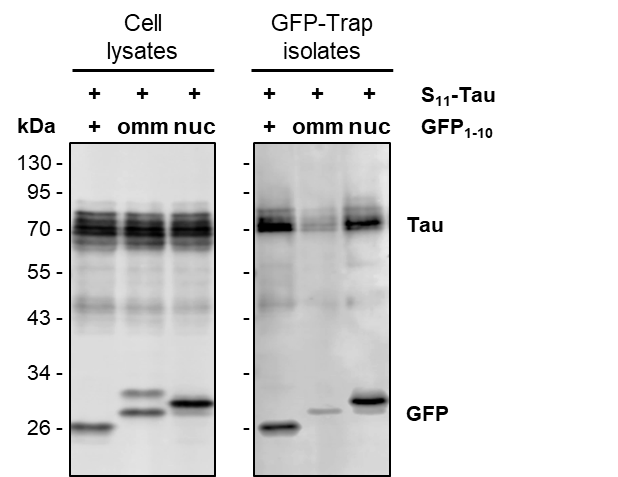


**Supplementary Figure 5**

Cell lysates from C17.2 cells transfected with S_11_-Tau and the indicated GFP_1-10_ sensors are analysed by western blot before (cell lysates) or after immune isolation on anti-GFP magnetic beads (GFP-Trap isolates). The presence of human Tau is verified with the Tau13 mouse pan-antibody and that of the sensor with an anti-GFP goat antibody by dual infrared fluorescence imaging. The amount of Tau isolated with the ommGFP_1-10_ sensor is lower than that isolated with the other two sensors in this experiment. Molecular weight markers are given on the left of the blots.


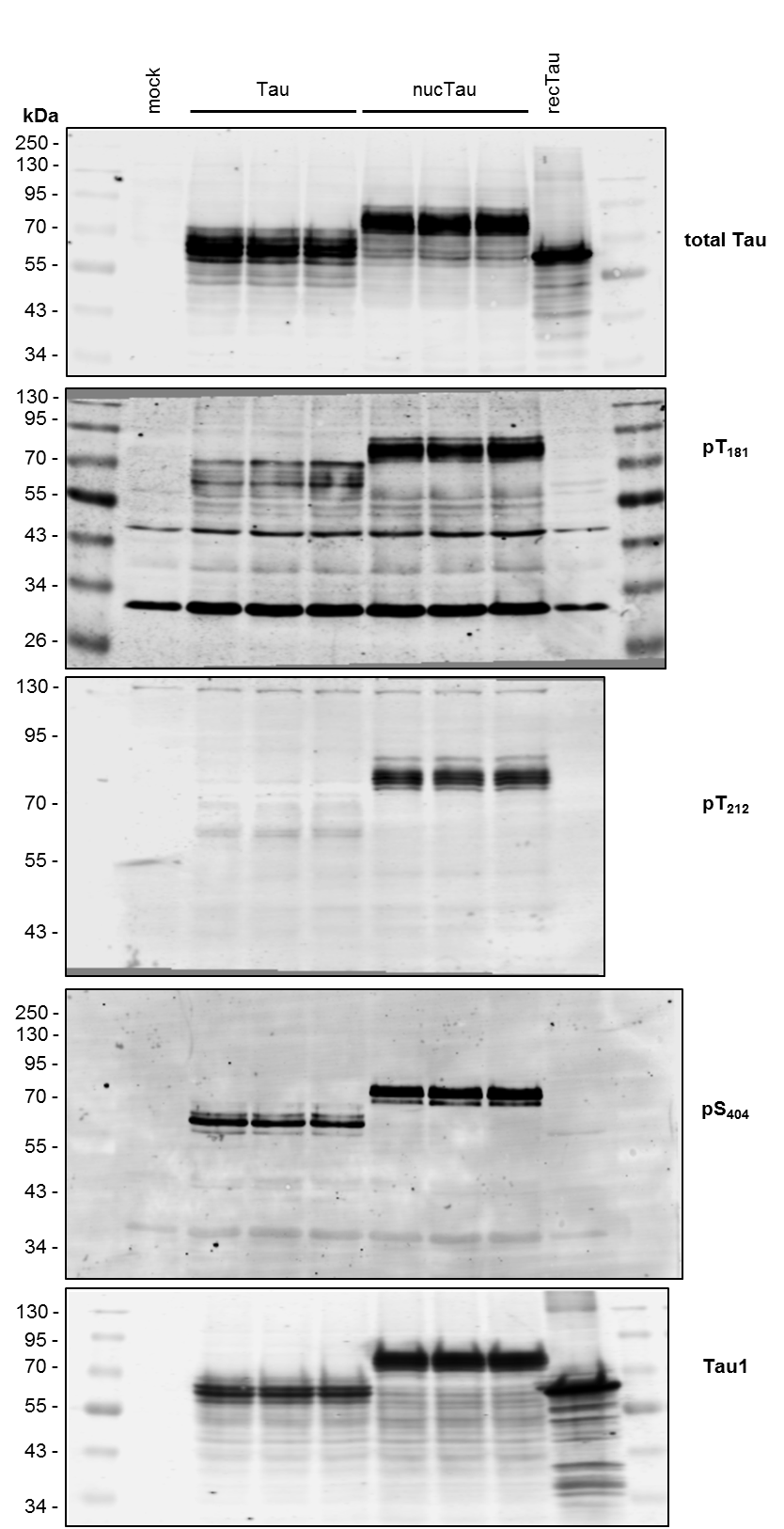


**Supplementary Figure 6**

Full western blots of the five panels shown in Fig. 5a (blotted with the five antibodies indicated and scanned by dual infrared fluorescence imaging). All samples were derived from the same experiment and processed in parallel.


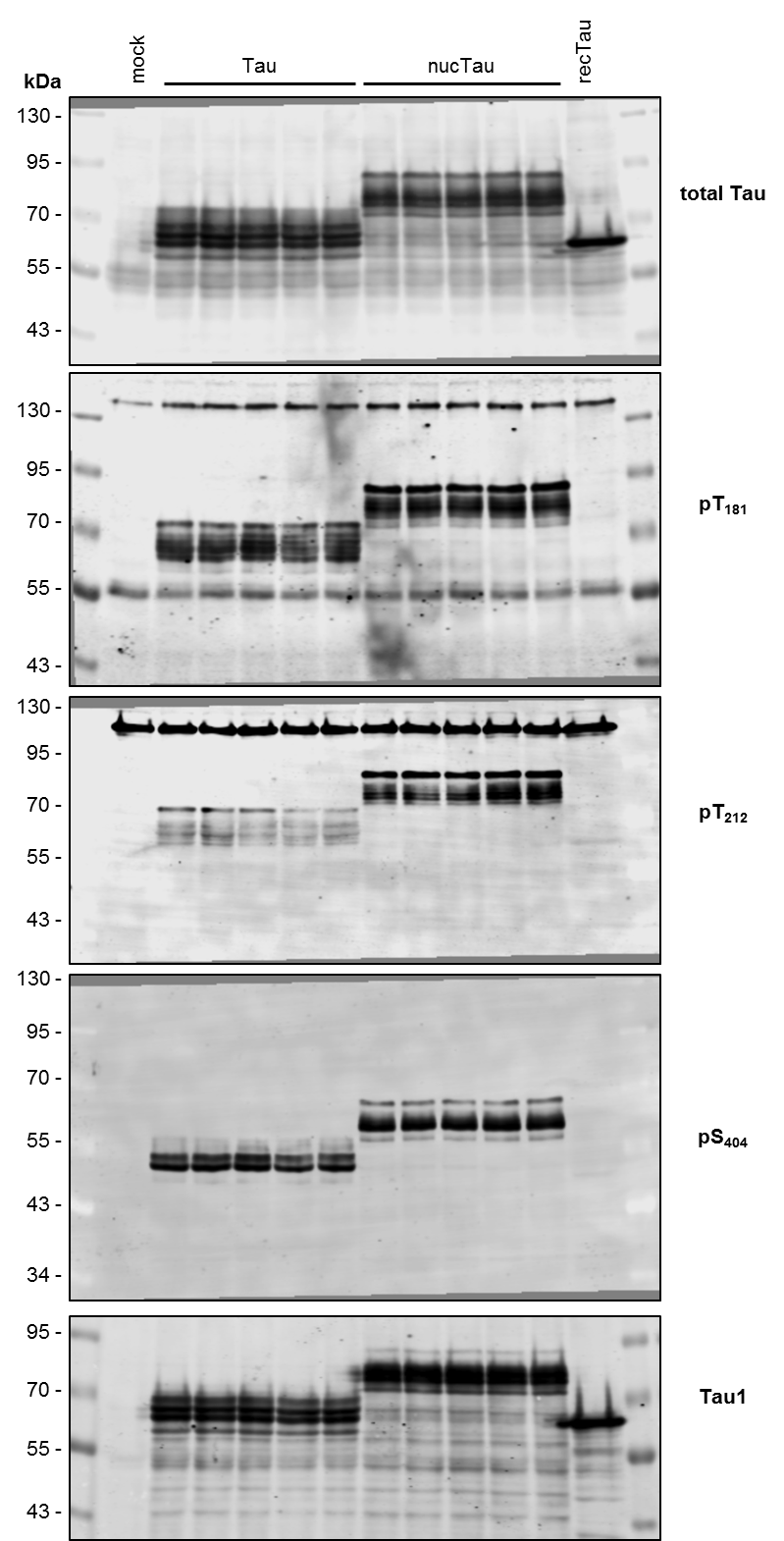


**Supplementary Figure 7**

Full western blots used for quantification shown in Fig. 5c (blotted with the five antibodies indicated and scanned by dual infrared fluorescence imaging). All samples were derived from the same experiment and processed in parallel.


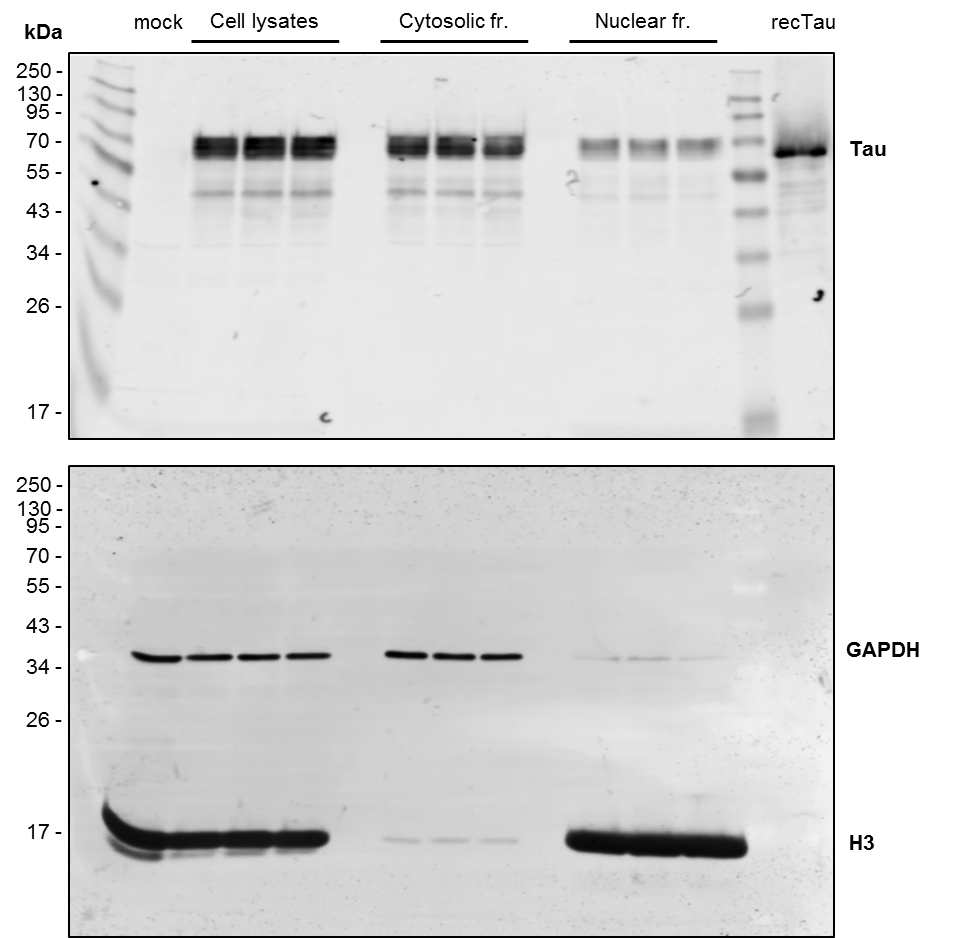


**Supplementary Figure 8**

Full western blot of the three panels shown in Fig. 6a (blotted with the three antibodies indicated and scanned by dual infrared fluorescence imaging). All samples were derived from the same experiment and processed in parallel.


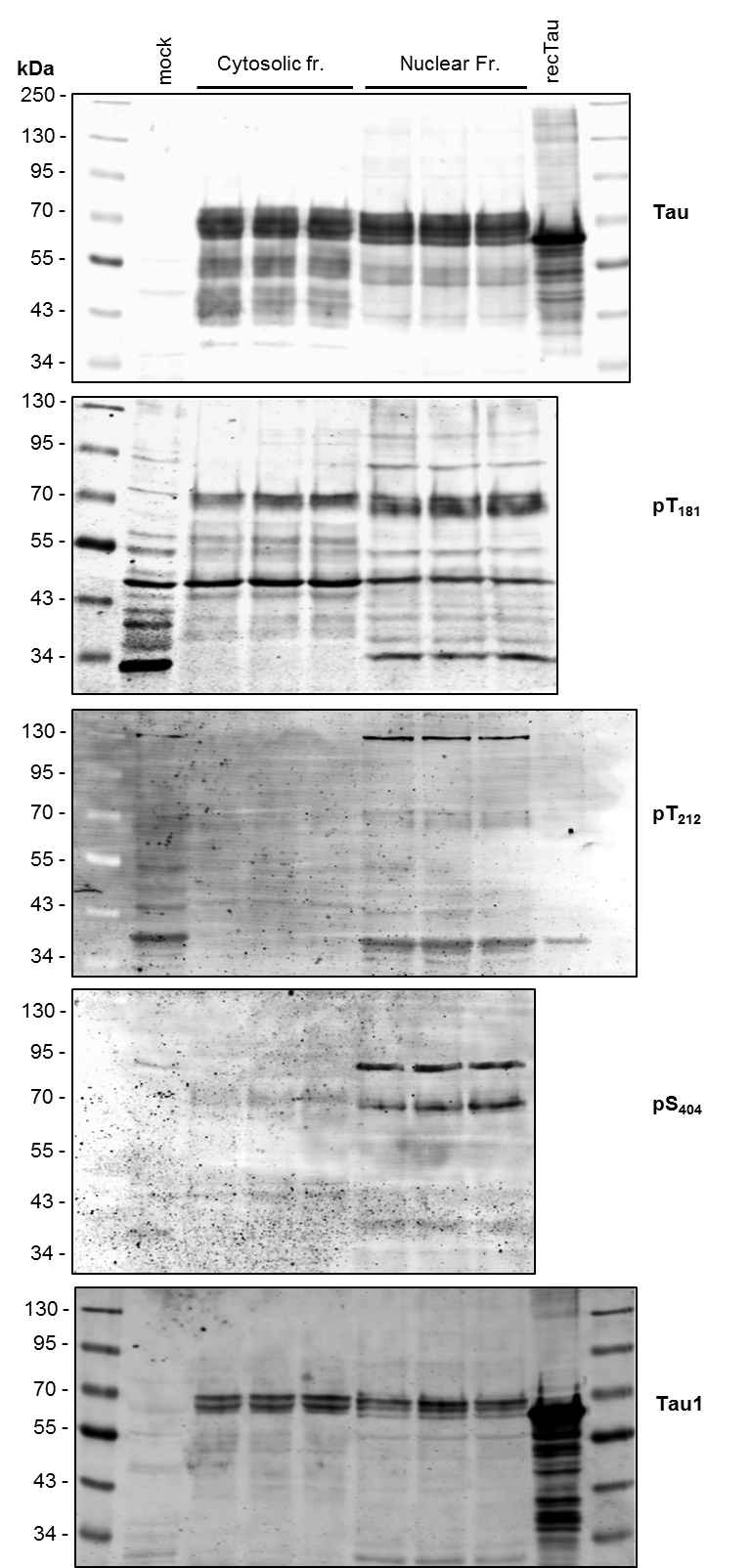


**Supplementary Figure 9**

Full western blots of the five panels shown in Fig. 6c (blotted with the five antibodies indicated and scanned by dual infrared fluorescence imaging). All samples were derived from two experiments and processed in parallel.

**
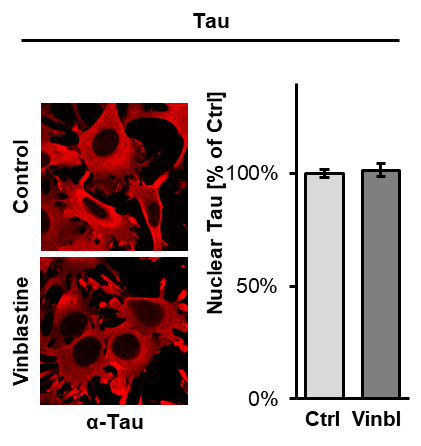
**

**Supplementary Figure 10**

Confocal microscopy images of C17.2 cells with induced Tau_441_ expression treated in the absence or presence of 3 µM Vinblastine for 5 hr and stained with the human Tau antibody (Tau13). Quantification of immune fluorescent detection of human Tau in DAPI-stained nuclei in the absence of presence of Vinblastine. Mean percent ± sem relative to the control. 2-tailed unpaired Mann-Whitney test, not significant.
